# Supplementary material for: Boron Clusters as Enhancers of RNase H Activity in the Smart Strategy of Gene Silencing by Antisense Oligonucleotides
Source: Int J Mol Sci. 2022 Oct 13;23(20):12190. doi: 10.3390/ijms232012190 (PMC9603397; doi:10.3390/ijms232012190)
Supplement: Supplementary file 1 [file ijms-23-12190-s001.zip › ijms-1943965-supplementary.pdf]

Supplementary Material

Boron clusters as enhancers of RNase H activity in the smart strategy of gene silencing by antisense oligonucleotides

Damian Kaniowski[1,*], Katarzyna Kulik[1], Justyna Suwara[1] , Katarzyna Ebenryter-Olbińska[1], Barbara Nawrot [1,*]

[1]*Centre of Molecular and Macromolecular Studies, Polish Academy of Sciences, Sienkiewicza 112, 90-363 Lodz, Poland*

* correspondence: dkanio@cbmm.lodz.pl; bnawrot@cbmm.lodz.pl

Table of contents:

1. **Spectral and mass spectrometry analysis of products**

   Figure S1. Analytical RP HPLC profiles and MALDI-TOF or ESI-Q-TOF mass spectra of 1 - 4, P1a - P1e, P2a-P2c, $1a_1 – 1a_3$, $1b_1$, $1c_1$, $1d_1$, $1e_1$, $2a_1$, $2b_1$, $2c_1$.
   Figure S2. Spectral analysis of 8-(5-azido-3-oxa-pentoxy)-3-iron-bis(1,2-dicarbollide) $[Fe(C_2B_9H_{11})_2]^-$ (azido derivative of FESAN).
   Figure S3. Spectral analysis of 1-(3-azidopropyl)-1,2-dicarba-*closo*-dodecaborane (1,2-DCDDB, $[C_2B_{10}H_{12}]$) (azido derivative of 1,2-dicarba-*closo*-dodecaborate).
   Figure S4. Spectral analysis of the bis-tetrabutylammonium-(4-azidobuthoxy)-undecahydro-*closo*-dodecaborate (DDB, $[B_{12}H_{12}]^{2-}$) (azido derivative of *closo*-dodecaborate).

Figure S1. Analytical RP HPLC profiles and MALDI-TOF or ESI-Q-TOF mass spectra of 1 - 4, P1a - P1e, P2a-P2c, $1a_1 – 1a_3$, $1b_1$, $1c_1$, $1d_1$, $1e_1$, $2a_1$, $2b_1$, $2c_1$.

RP-HPLC conditions were as follow: the buffer A (0.1 M CH_3COONH_4) and the buffer B (100% CH_3CN). The buffer B gradient:
B1: 0→2 min 0%; 2→25 min 0-48%; 25→30 min 48-60%; 30→35 min 60-0%; 35→38 min 0%
B2: 0→2 min 0%; 2→25 min 0-45%; 25→28 min 48-60%; 28→30 min 60-0%; 30→33 min 0%.
RP-HPLC conditions for **3** and **4** were as follow: the buffer A (0.1 M CH_3COONH_4) and the buffer B(0.1 M CH_3COONH_4 / 40%CH_3CN). The buffer B3 gradient: 0→3 min 0%; 3→23 min 0-100%; 23→27 min 100%; 27→30 min 100-0%; 30→32 min 0%.

1: 5′-d(TTT CTT TTC CTC C)-3′

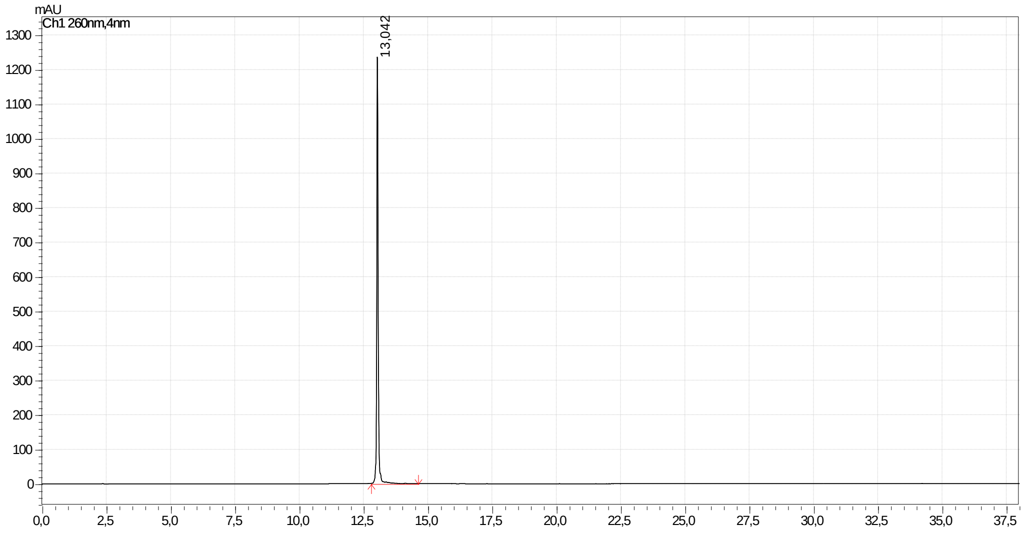

ESI-Q-TOF

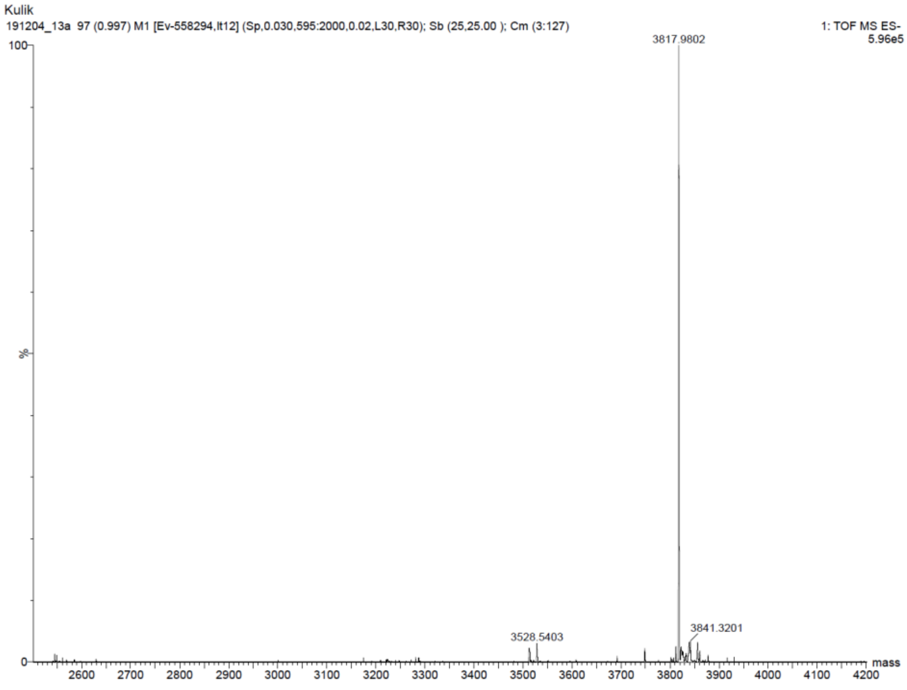

2: 5′-d(CTC CAG AGC CCG A)-3′

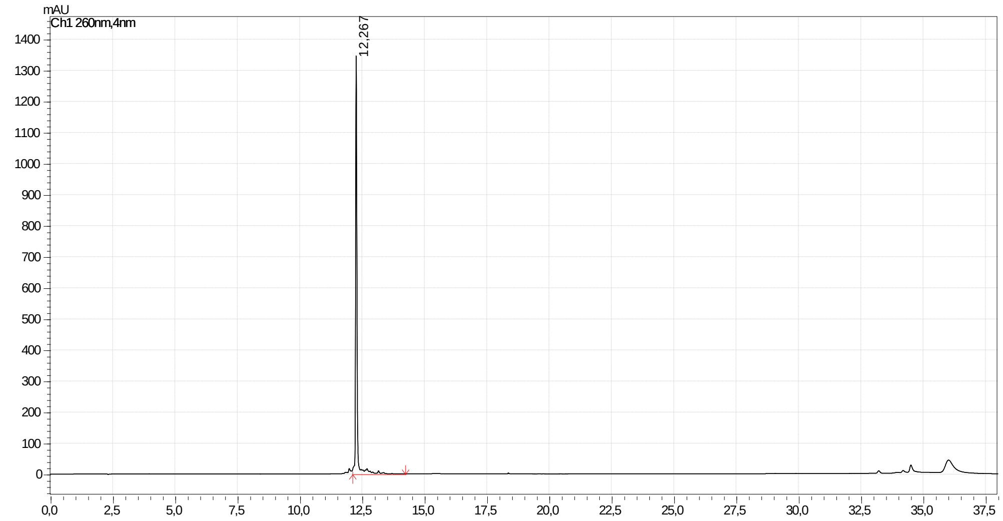

ESI-Q-TOF

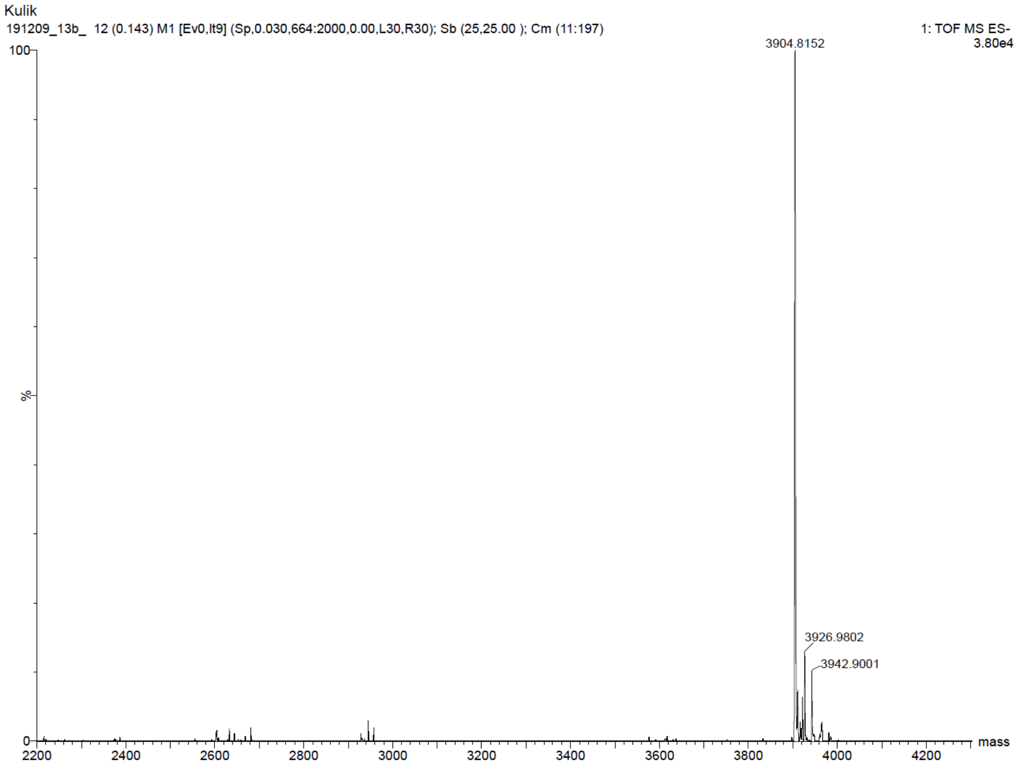

P1a: 5′-d(UPrTT CTT TTC CTC C)-3′

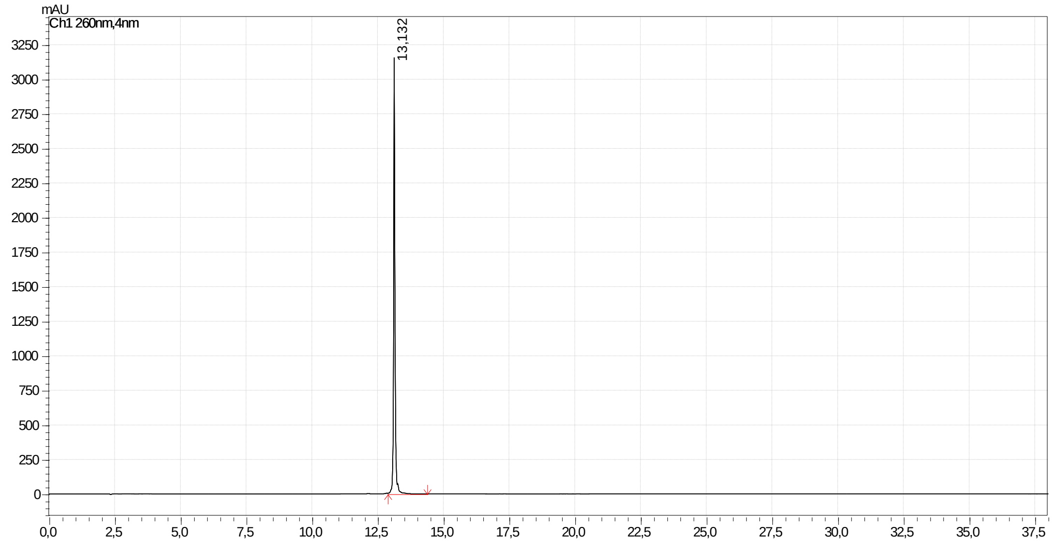

ESI-Q-TOF

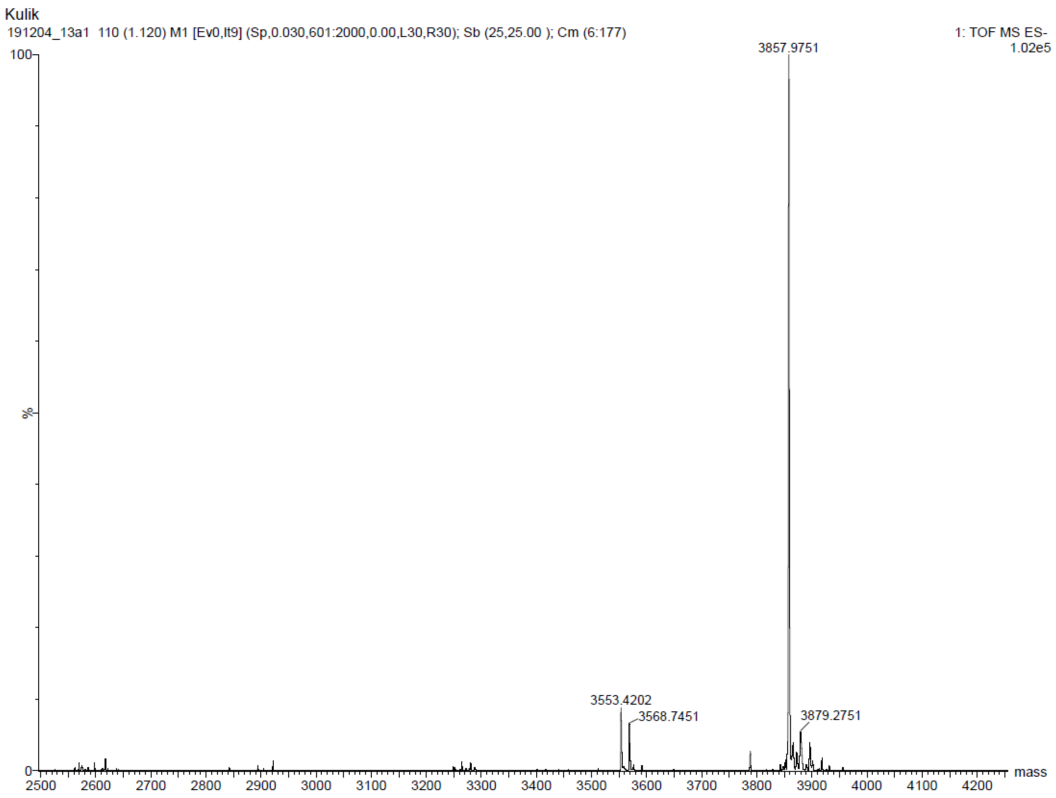

P1b: 5′-d(TTU_{Pr} CTT TTC CTC C)-3′

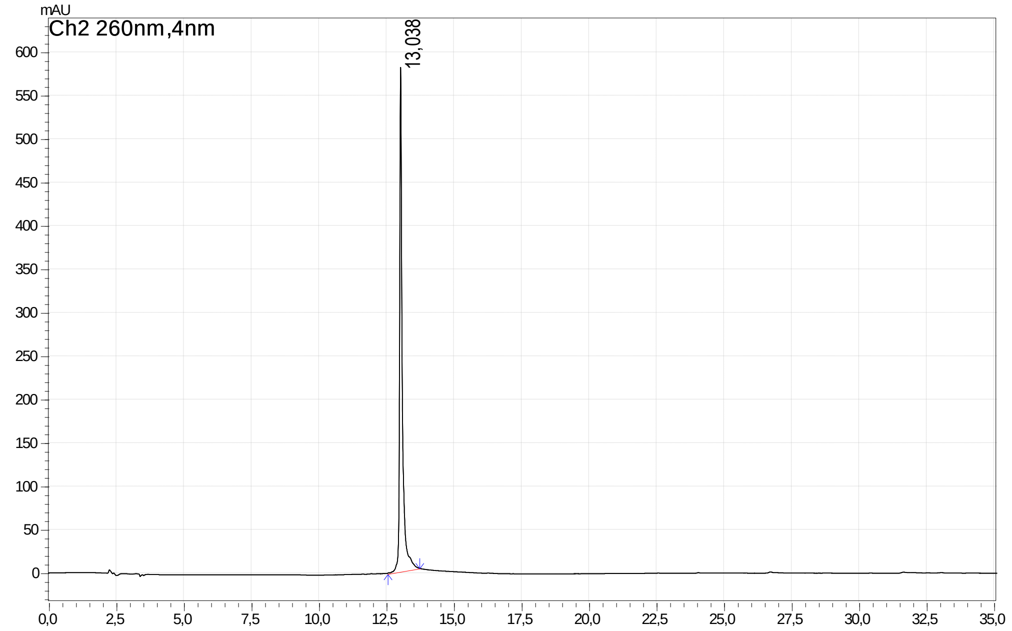

MALDI-TOF

Ebenryter-Olbinska.K, 13a3, linear neg
HPA 50 mg/mL H2O/ACN 1:1 v/v, AC 50 mg/mL H2O/ACN 1:1 v/v; HPA/AC 8:1
Data: hg770002.D2[c] 13 Mar 2019 15:11 Cal: HPA_T5_T124 31 Oct 2018 15:37
Shimadzu Biotech Axima Performance 2.9.1.20100121: Mode Linear_neg_2018, Power: 104, Blanked, P.Ext. @ 2800 (bin 76)
%Int. 13 mV[sum= 1411 mV] Profiles 1-107 Smooth Gauss 15 -Baseline 60

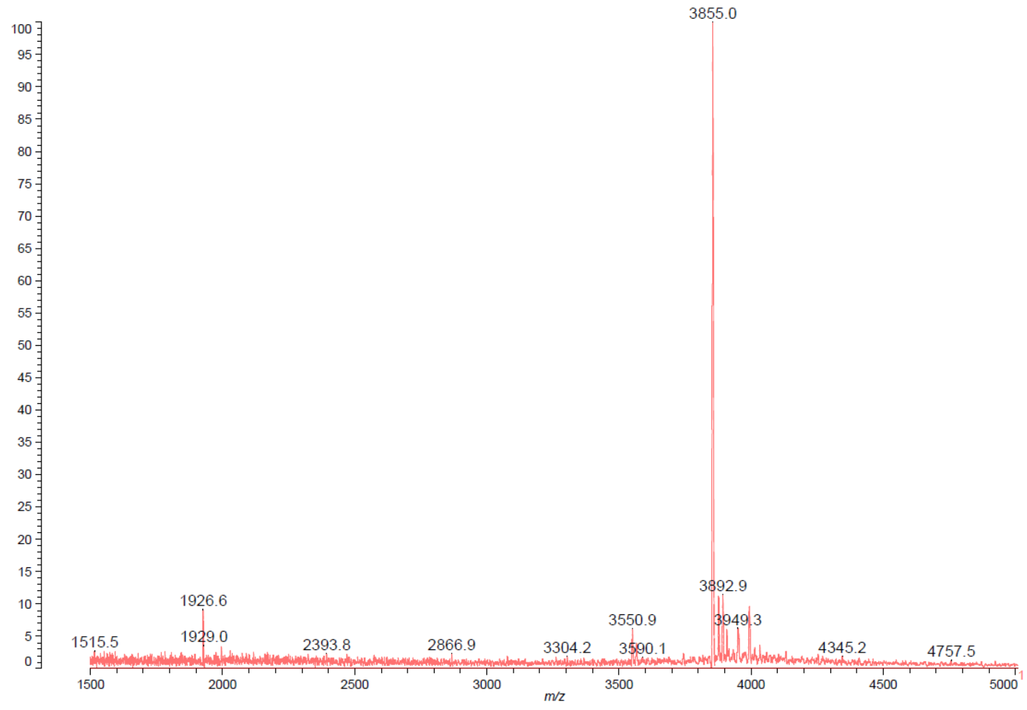

P1c: 5′-d(TTT CTT U_{Pr}TC CTC C)-3′

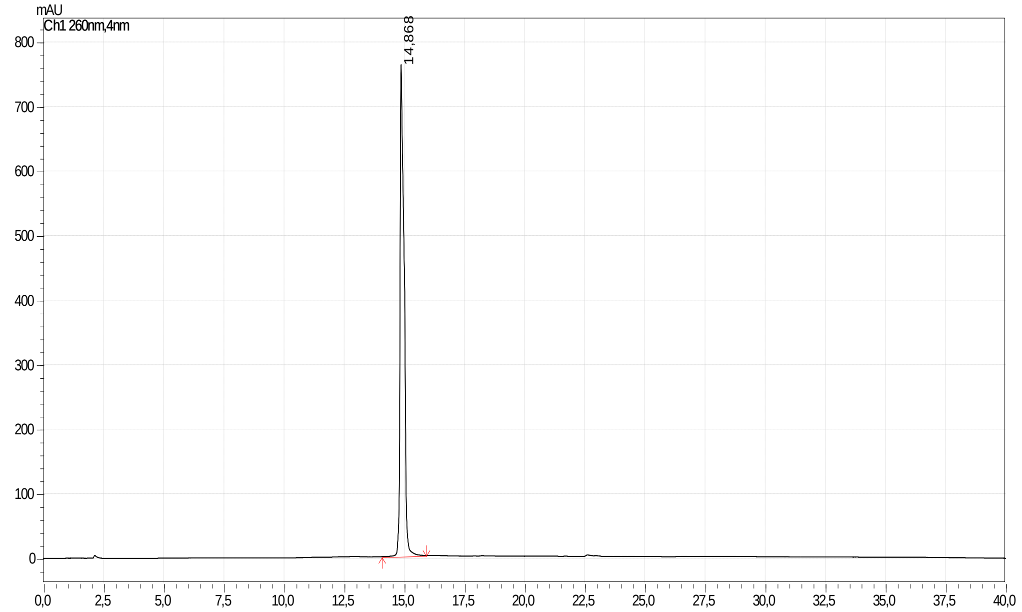

ESI-Q-TOF

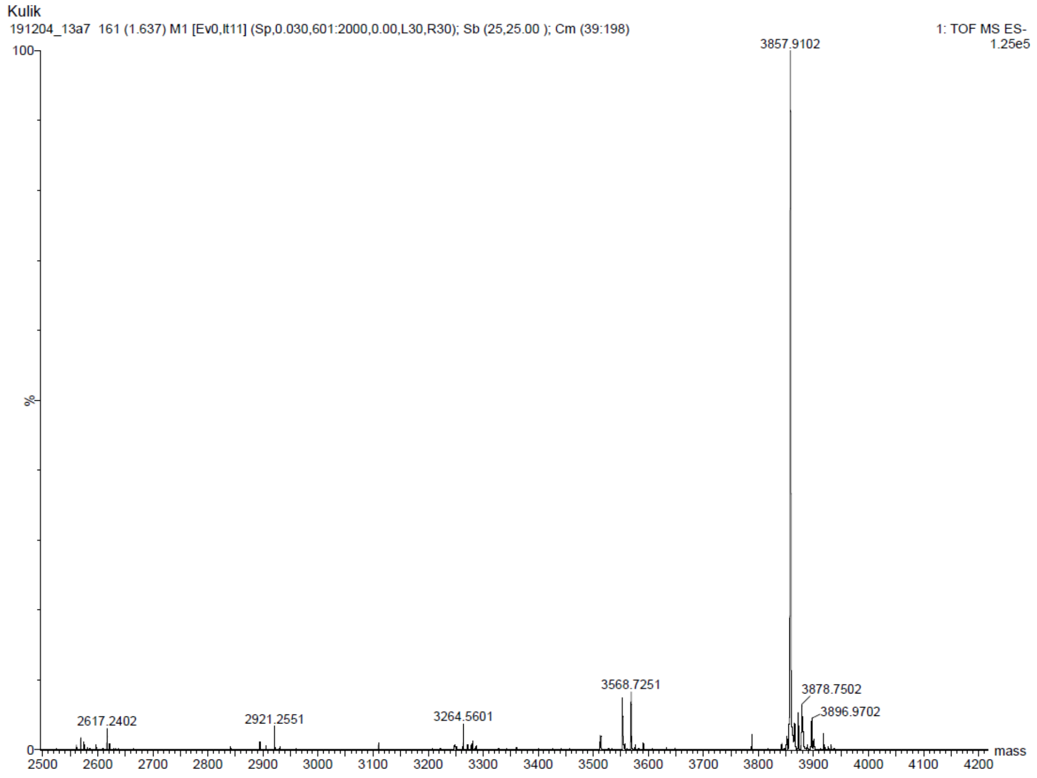

P1d: 5′-d(TTT CTT TTC CU$_{Pr}$C C)-3′

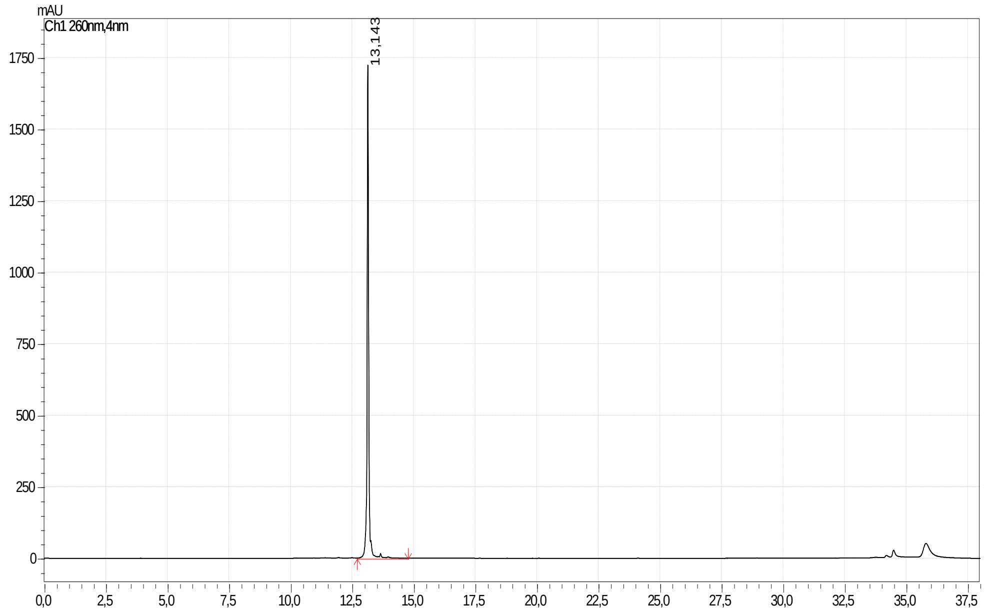

ESI-Q-TOF

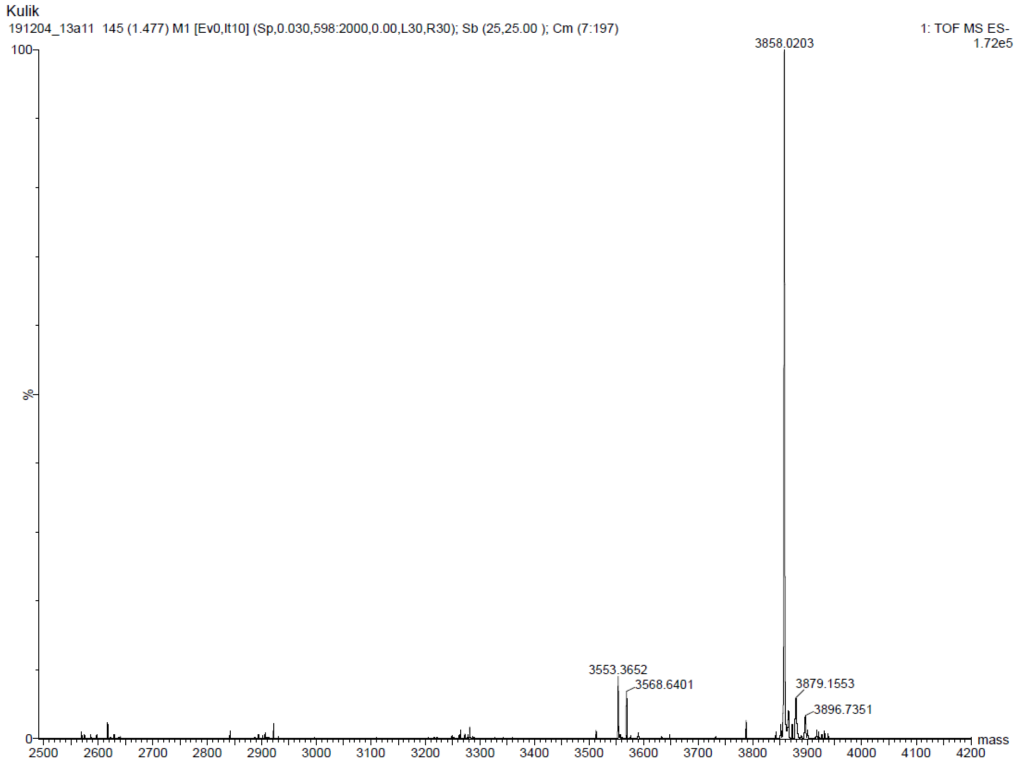

P1e: 5′-d(TTU$_{Pr}$ CTT U$_{Pr}$TC CTC C)-3′

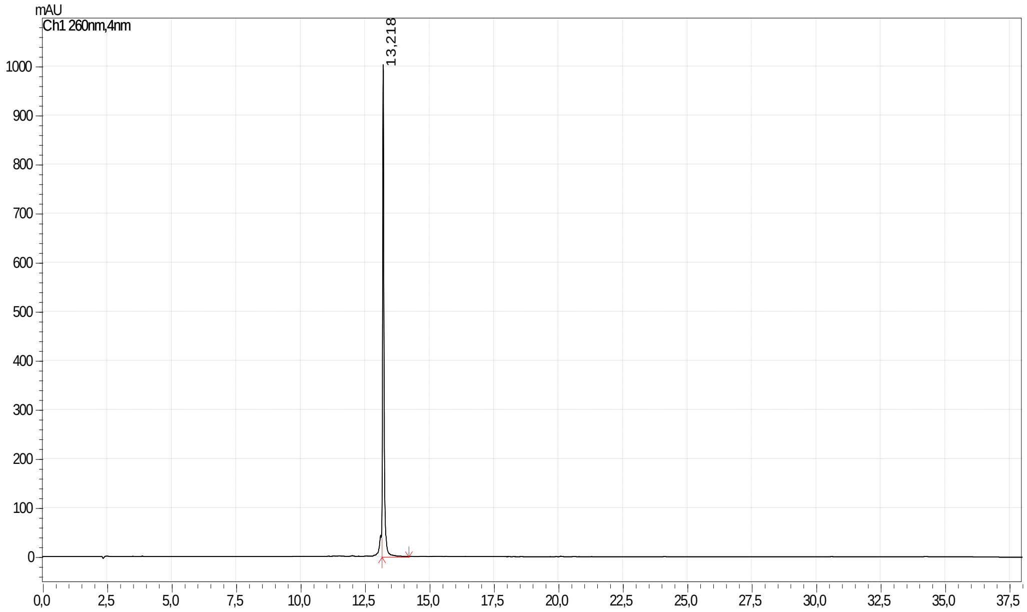

ESI-Q-TOF

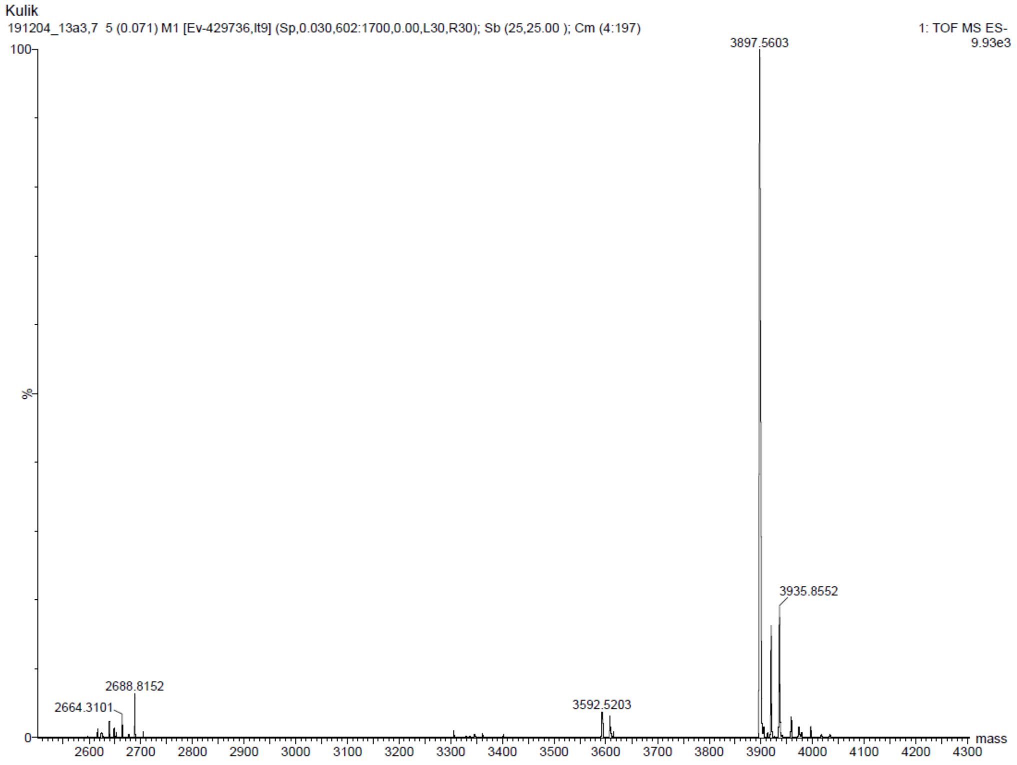

P2a: 5′-d(CU$_{Pr}$C CAG AGC CCG A)-3′

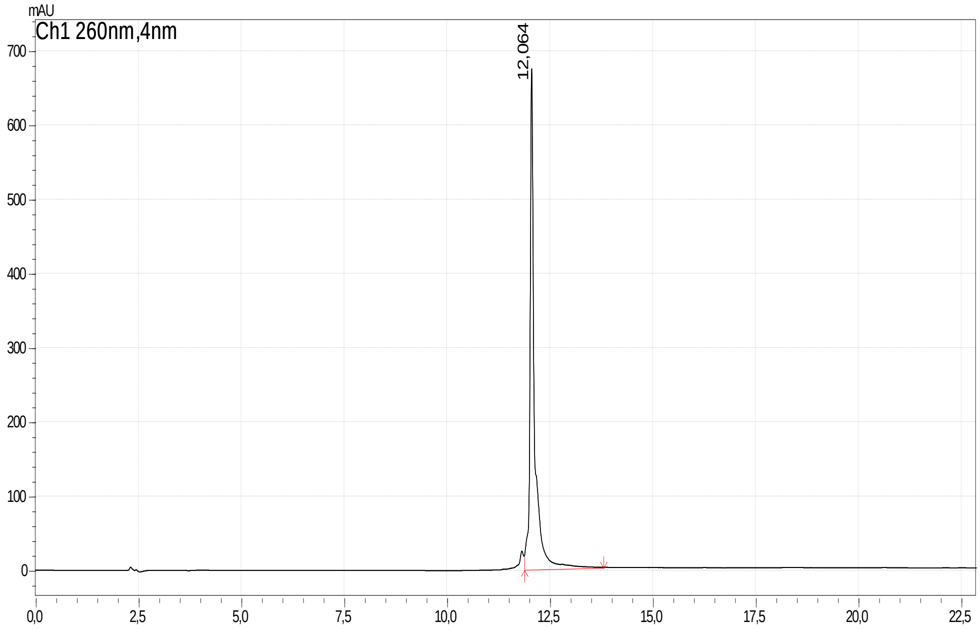

ESI-Q-TOF

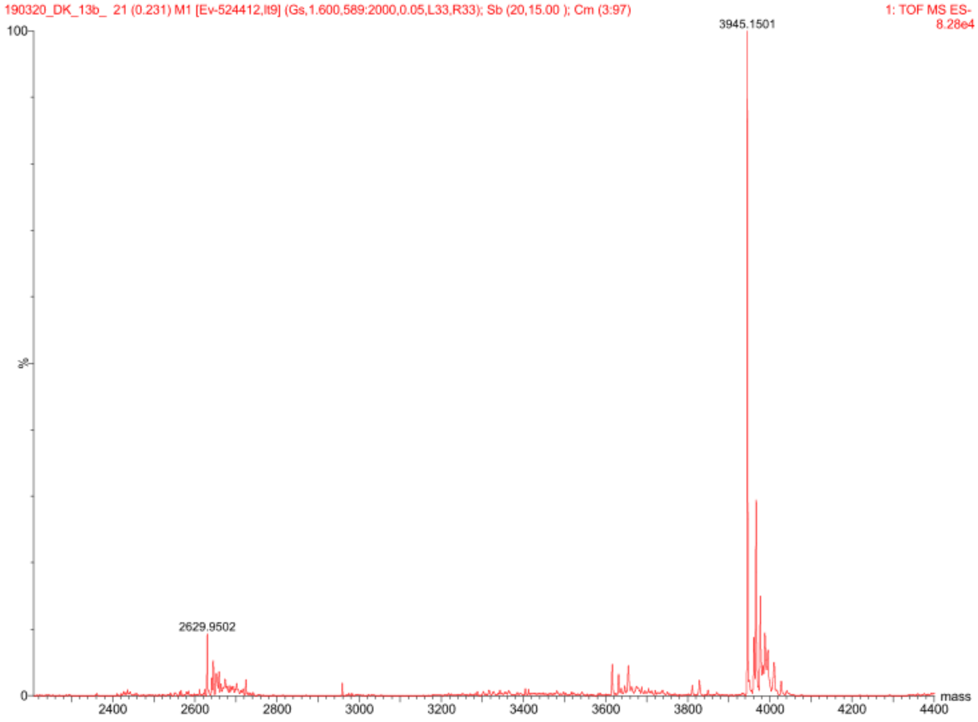

P2b: 5′-d(CTC CAG AGC CU_{Pr}G A)-3′

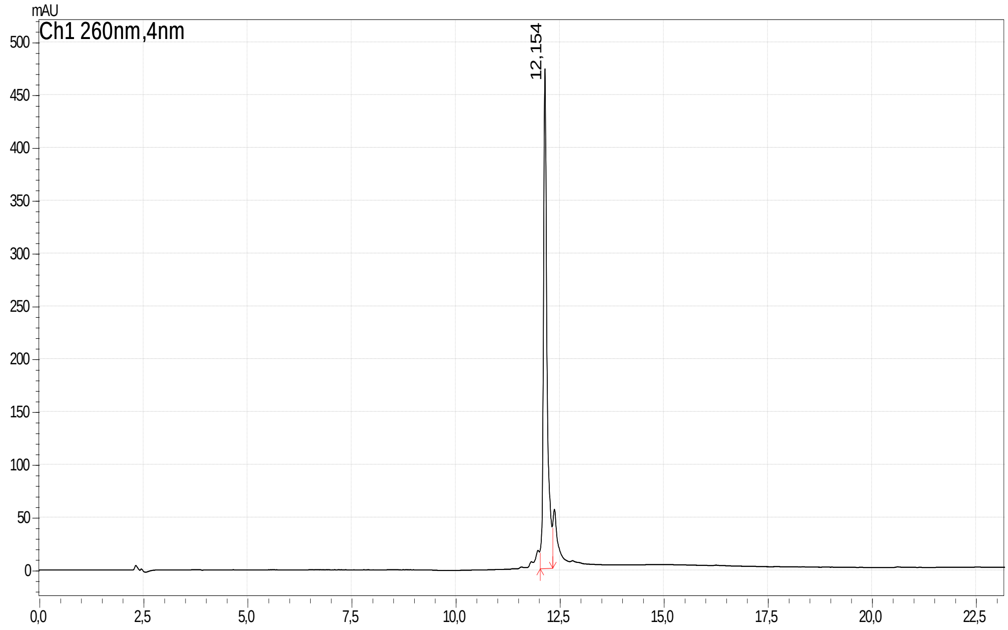

MALDI-TOF

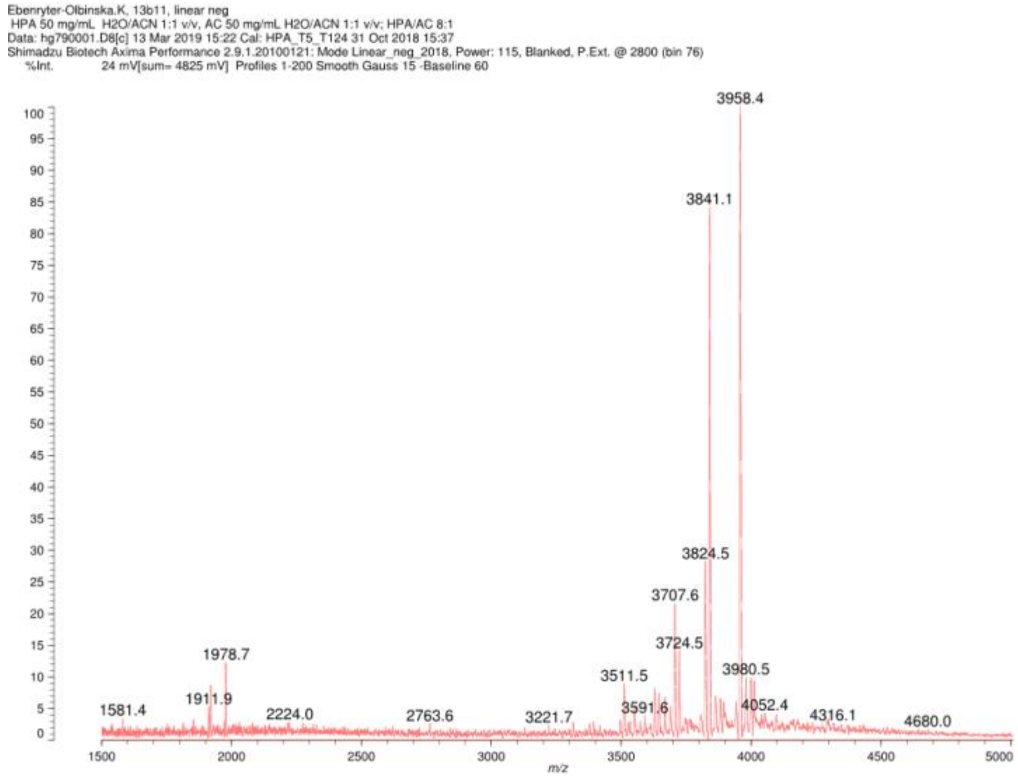

P2c: 5′-d(CU_{Pr}C CAG AGC CU_{Pr}G A)-3′

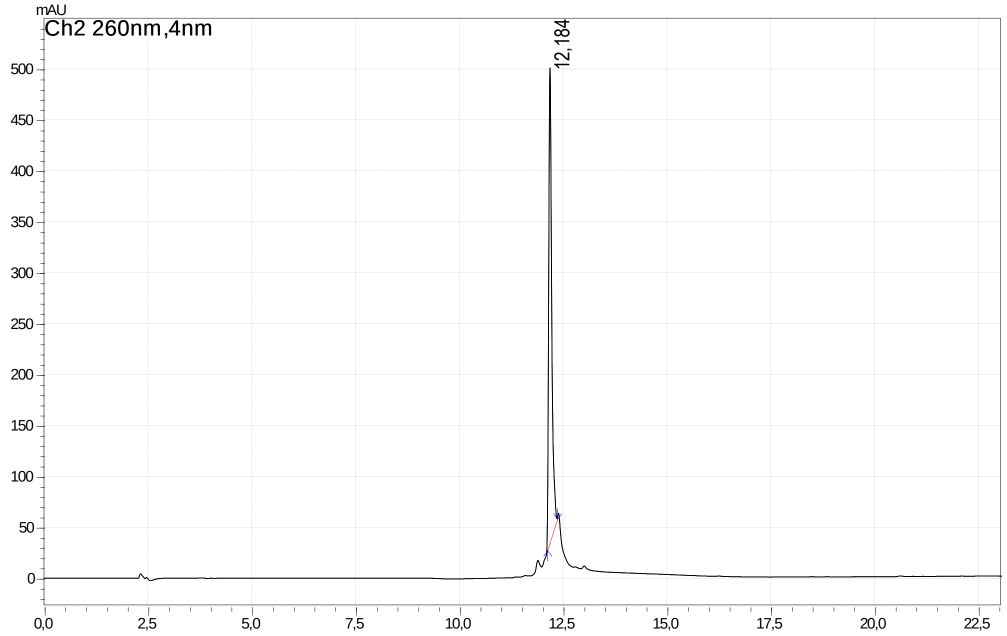

MALDI-TOF

Ebenryter-Olbinska.K, 13b2,11, linear neg
HPA 50 mg/mL H2O/ACN 1:1 v/v, AC 50 mg/mL H2O/ACN 1:1 v/v; HPA/AC 8:1
Data: hg780004.D6[c] 13 Mar 2019 15:20 Cal: HPA_T5_T124 31 Oct 2018 15:37
Shimadzu Biotech Axima Performance 2.9.1.20100121: Mode Linear_neg_2018, Power: 109, Blanked, P.Ext. @ 2800 (bin 76)
%Int. 12 mV[sum= 1475 mV] Profiles 1-128 Smooth Gauss 15 -Baseline 60

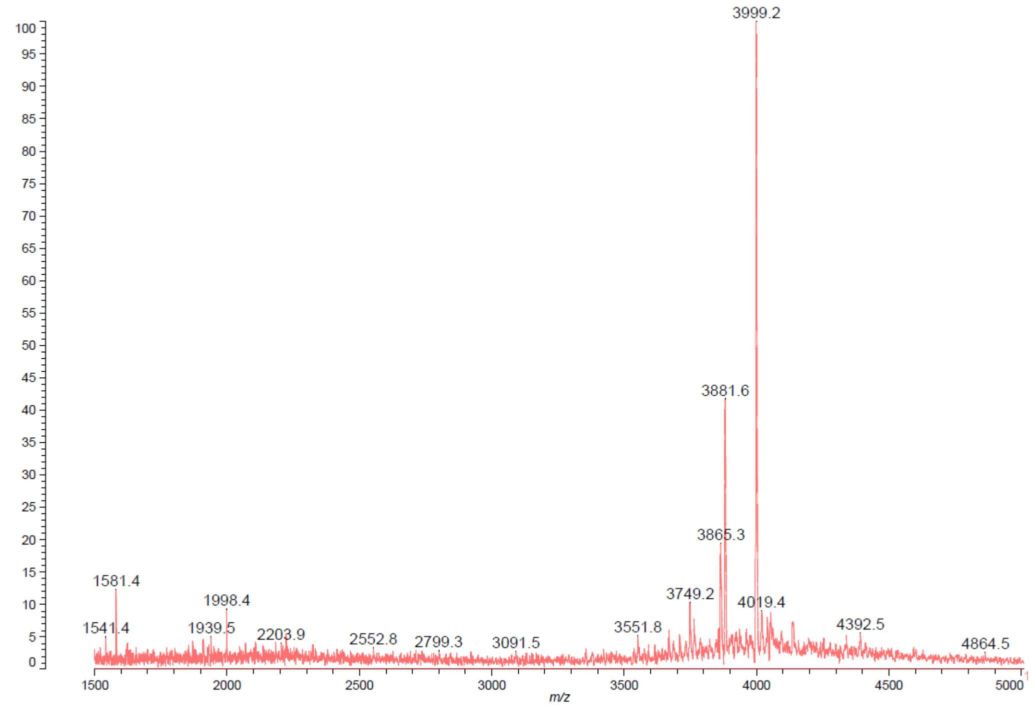

1a$_1$: 5′-d(U$_{B1}$TT CTT TTC CTC C)-3′

Datafile Name:11.12.18 KK321 analit 0,05 OD1.lcd
Sample Name:11.12.18 KK321 analit 0,05 OD
Sample ID:13.04.18

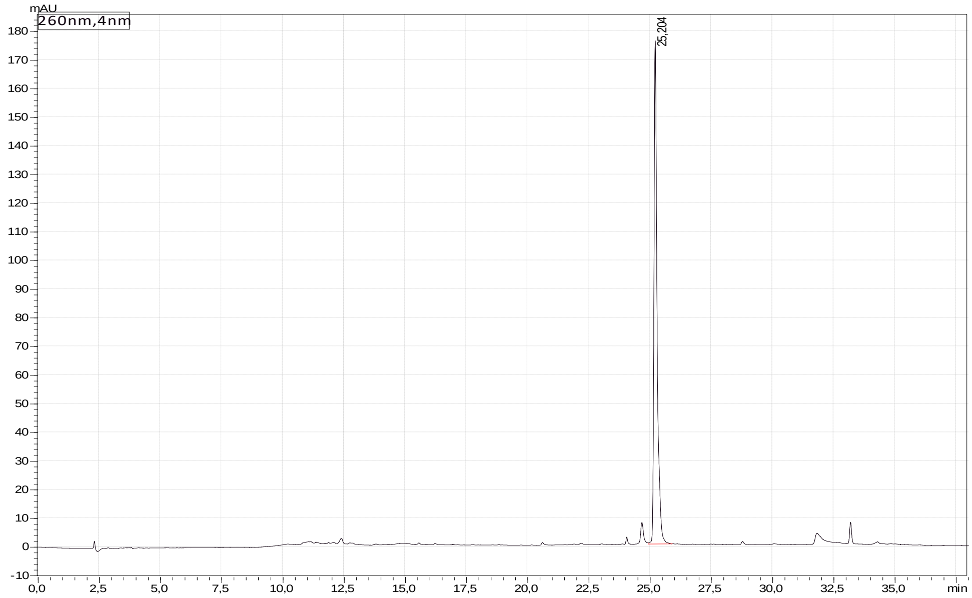

MALDI-TOF

Kulik K, KK-321, linear neg
HPA 50 mg/mL H2O/ACN 1:1 v/v, AC 50 mg/mL H2O/ACN 1:1 v/v; HPA/AC 8:1
Data: he280003.L11[c] 3 Dec 2018 15:03 Cal: HPA_T5_T18 31 Oct 2018 15:33
Shimadzu Biotech Axima Performance 2.9.1.20100121: Mode Linear_neg_2018, Power: 118, Blanked, P.Ext. @ 3800 (bin 88)
%Int. 33 mV[sum= 6576 mV] Profiles 1-200 Smooth Av 20 -Baseline 60

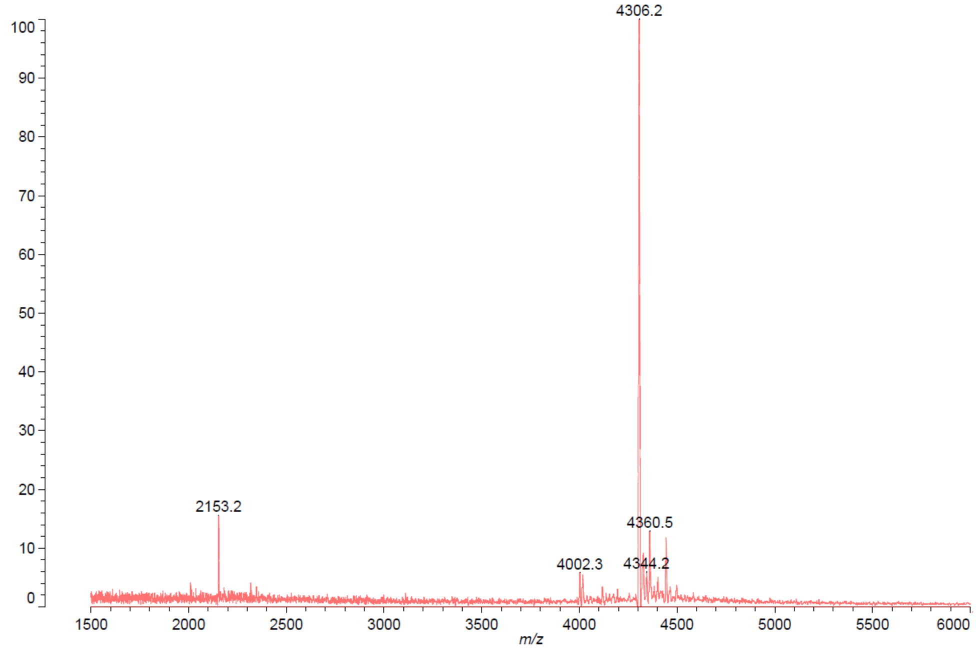

1a2: 5′-d(UB2TT CTT TTC CTC C)-3′ (B2 RP-HPLC program)

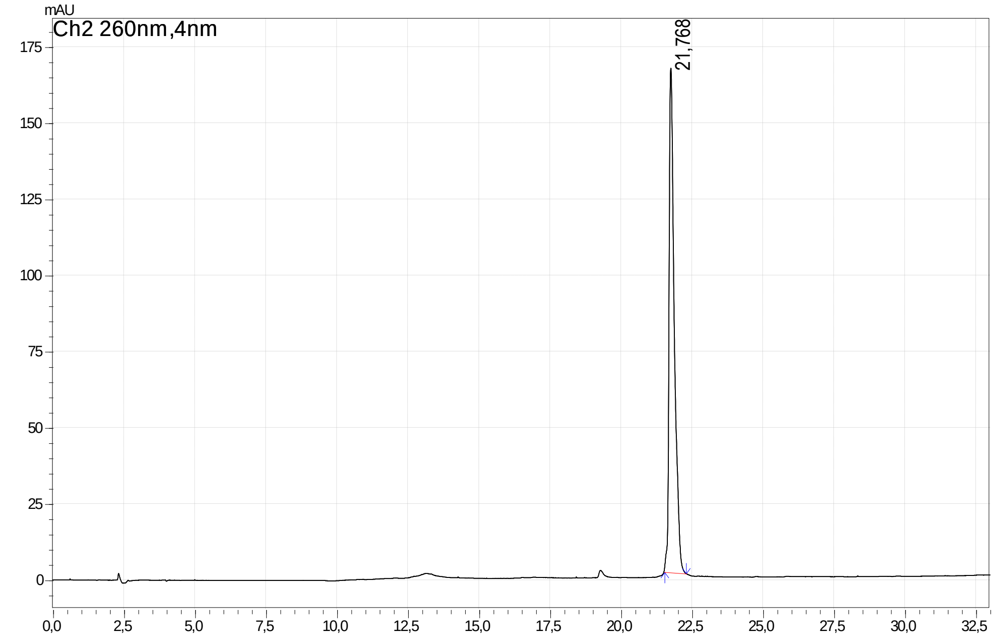

ESI-Q-TOF

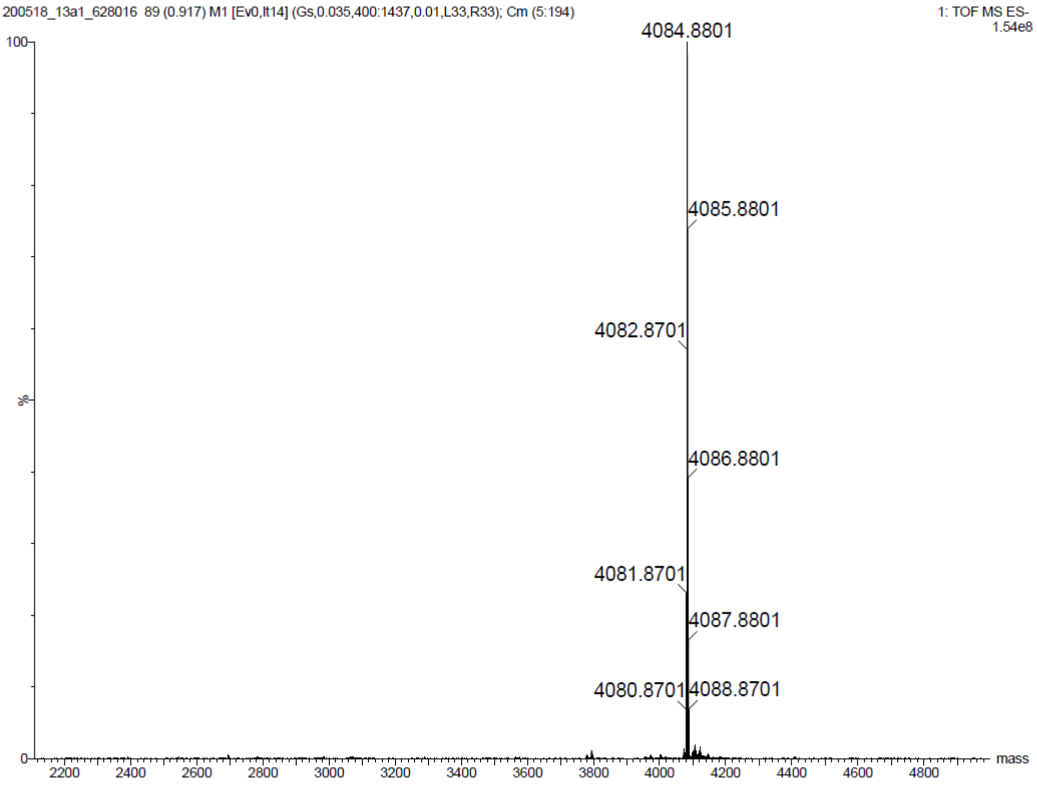

1a3: 5′-d(UB3TT CTT TTC CTC C)-3′ (B2 RP-HPLC program)

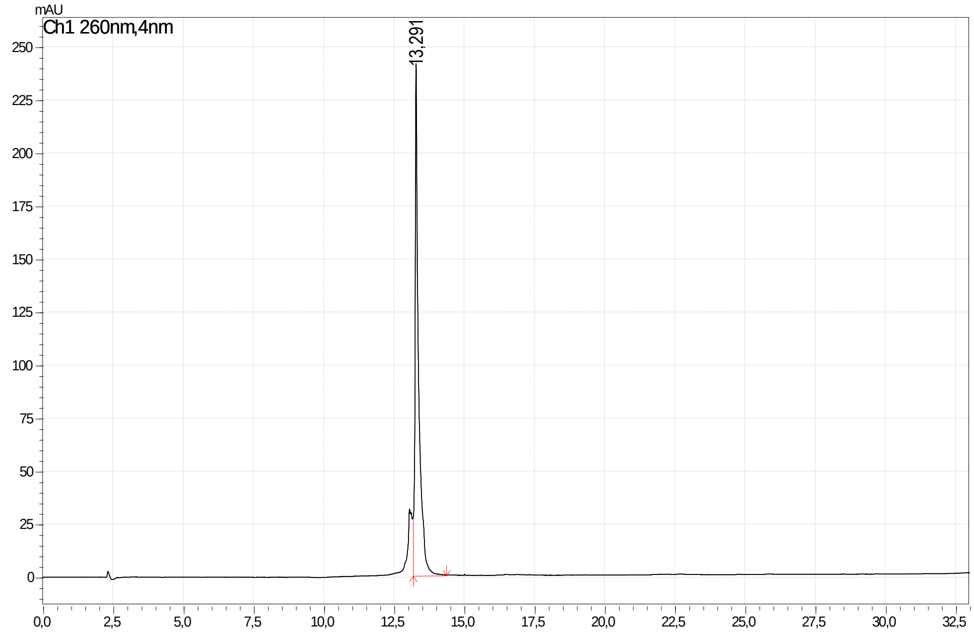

ESI-Q-TOF

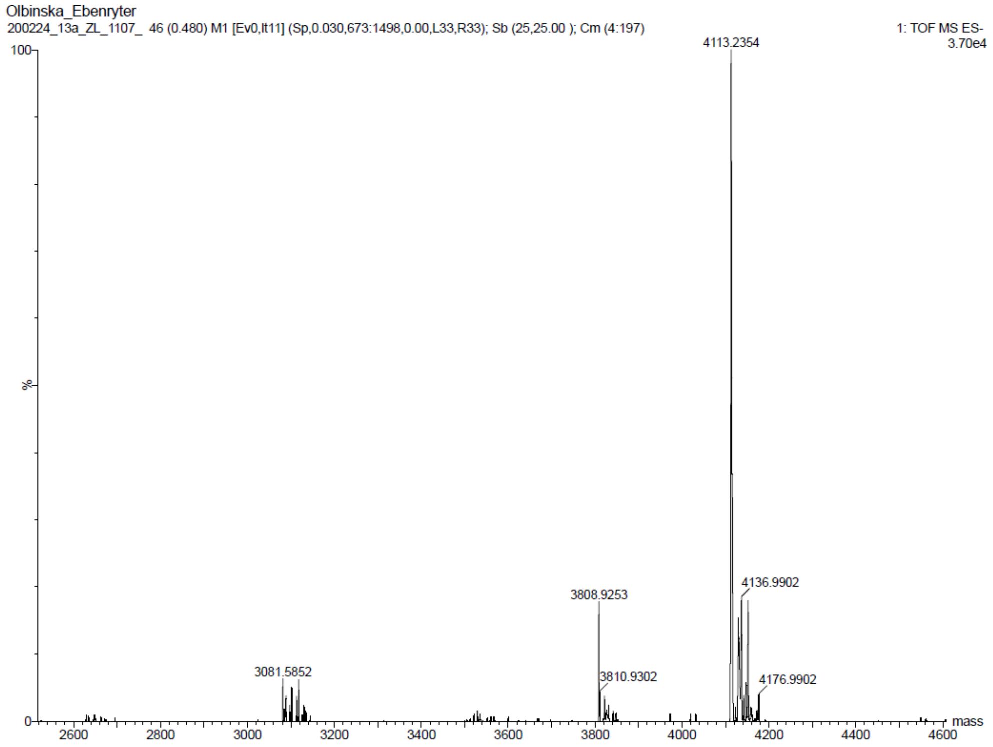

1b$_{1}$: 5′-d(TT U$_{B1}$ CTT TTC CTC C)-3′ (B2 RP-HPLC program)

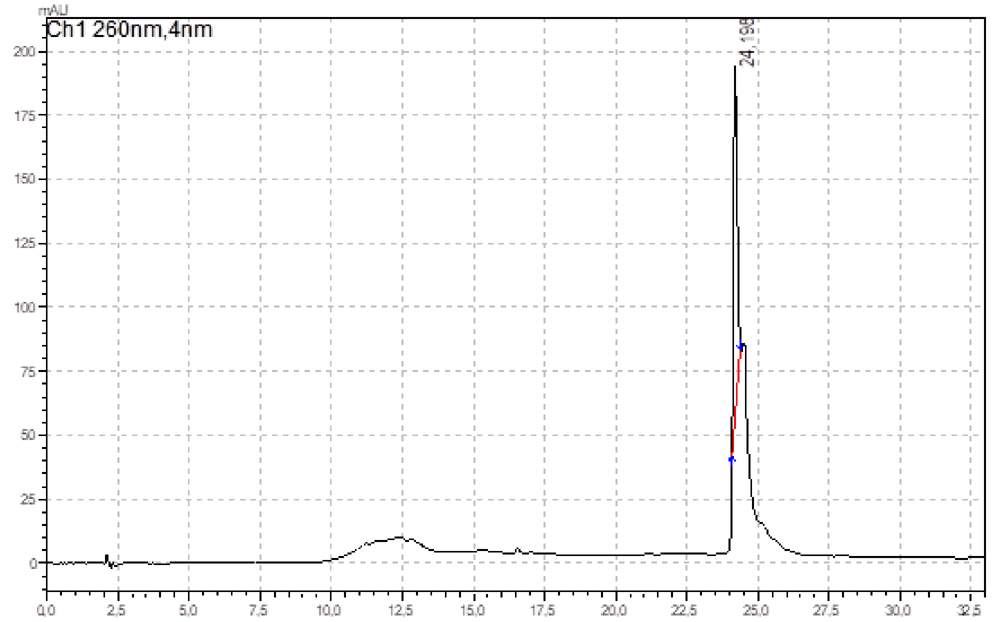

ESI-Q-TOF

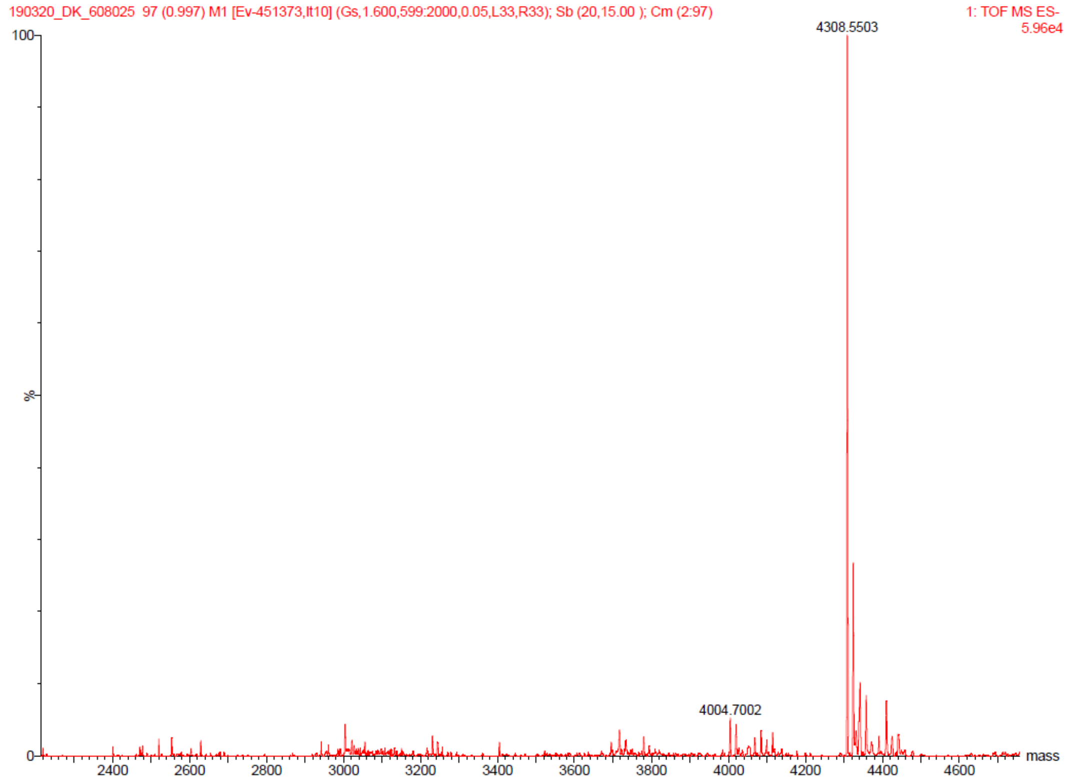

1c1 : 5′-d(TTT CTT UB1TC CTC C)-3′

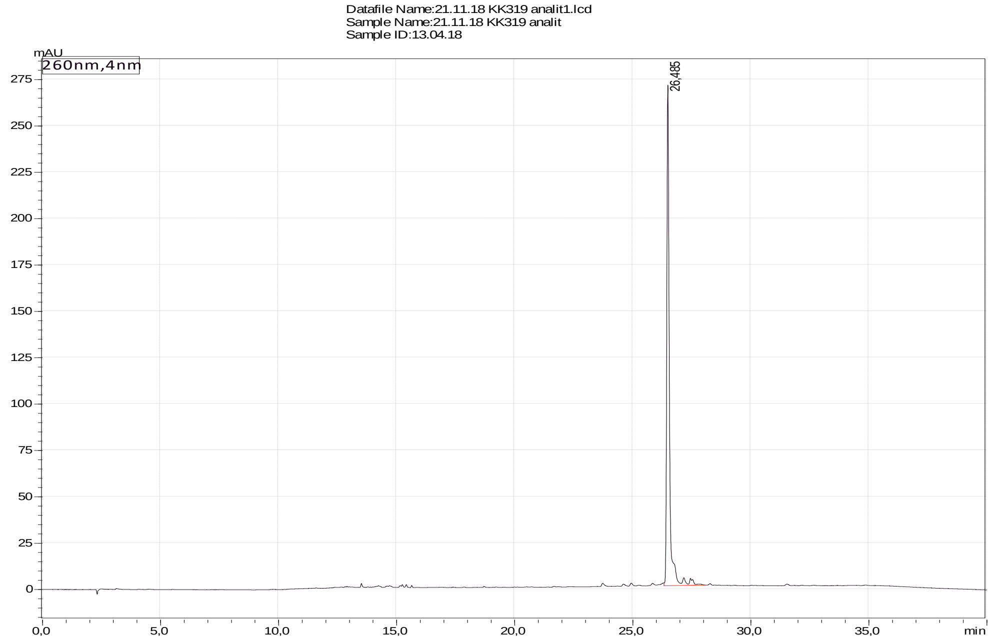

MALDI-TOF

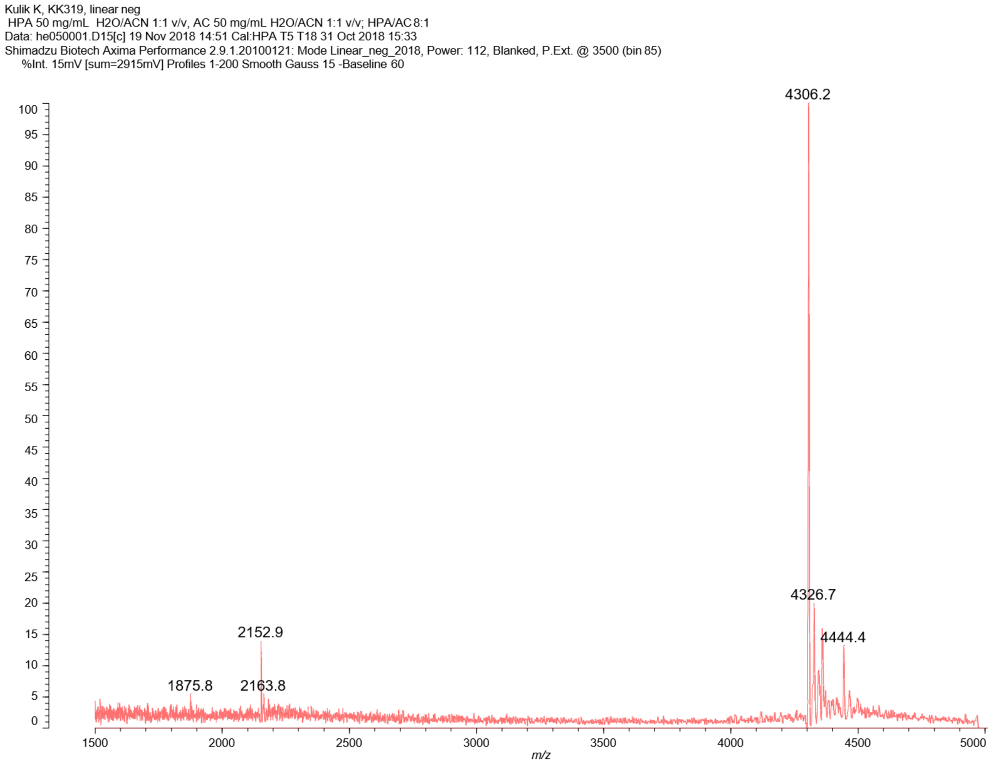

1d1: 5′-d(TTT CTT TTC CUB1C C)-3′

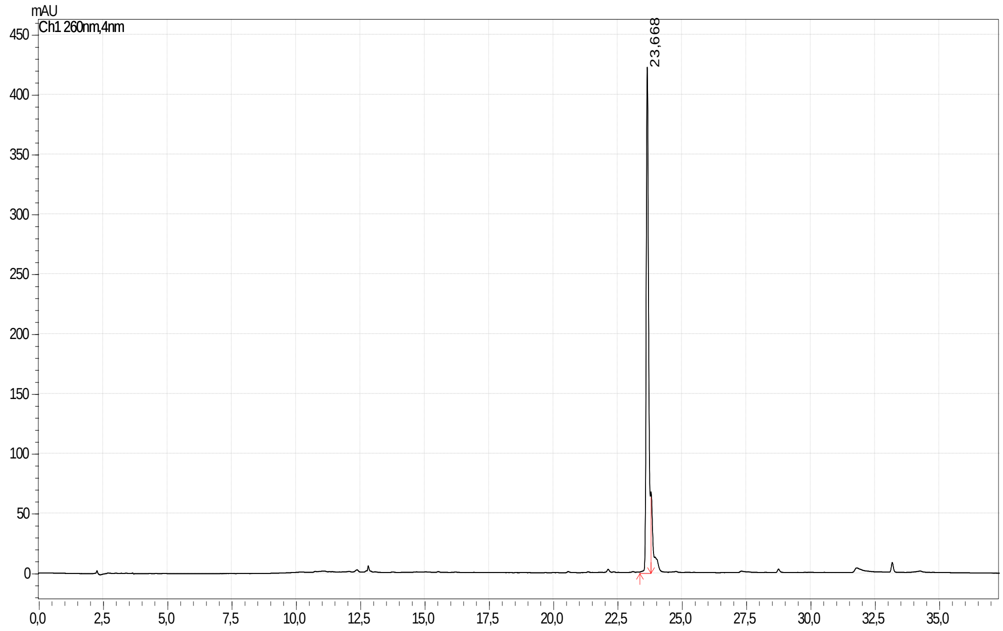

MALDI-TOF

Kulik K, KK-324, linear neg
HPA 50 mg/mL H2O/ACN 1:1 v/v, AC 50 mg/mL H2O/ACN 1:1 v/v; HPA/AC 8:1
Data: he290002.L13[c] 3 Dec 2018 15:08 Cal: HPA_T5_T18 31 Oct 2018 15:33
Shimadzu Biotech Axima Performance 2.9.1.20100121: Mode Linear_neg_2018, Power: 112, Blanked, P.Ext. @ 3500 (bin 85)
%Int. 65 mV[sum= 9830 mV] Profiles 1-152 Smooth Av 20 -Baseline 60

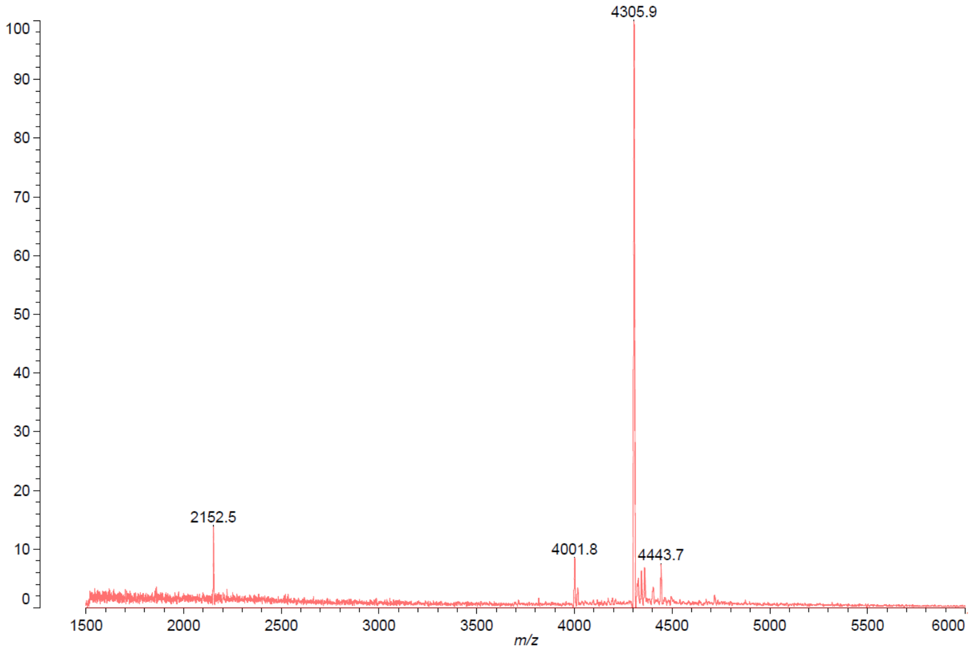

1e1: 5′-d(TTUB1 CTT UB1TC CTC C)-3′

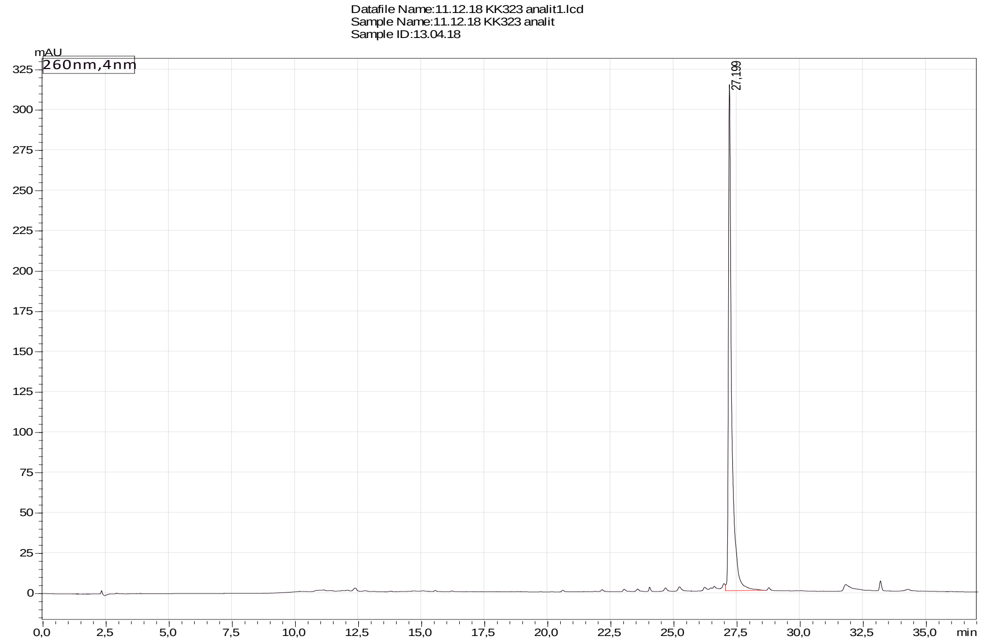

MALDI-TOF

Kulik K, KK-323, linear neg
HPA 50 mg/mL H2O/ACN 1:1 v/v, AC 50 mg/mL H2O/ACN 1:1 v/v; HPA/AC 8:1
Data: he310002.L17[c] 3 Dec 2018 15:16 Cal: HPA_T5_T18 31 Oct 2018 15:33
Shimadzu Biotech Axima Performance 2.9.1.20100121: Mode Linear_neg_2018, Power: 113, Blanked, P.Ext. @ 3500 (bin 85)
%Int. 21 mV[sum= 3053 mV] Profiles 1-142 Smooth Av 20 -Baseline 60

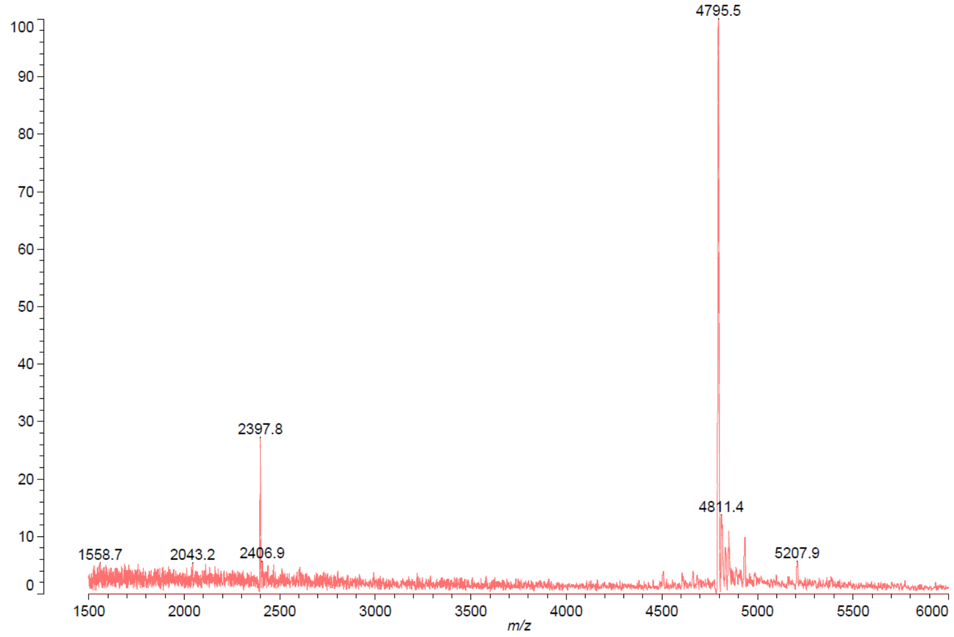

2a$_1$: 5′-d(CU$_{B1}$C CAG AGC CCGA)-3′

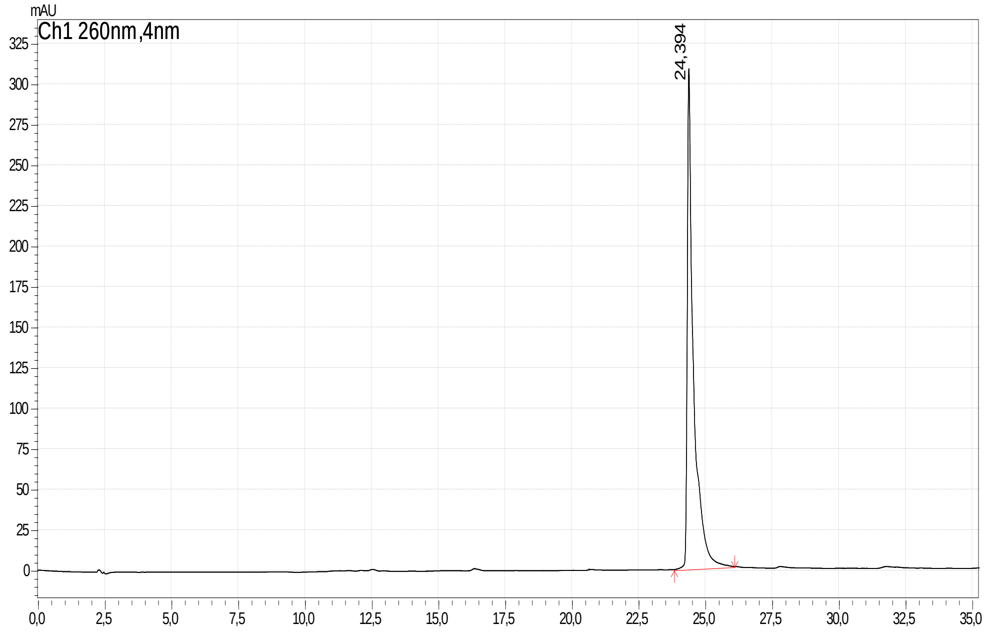

MALDI-TOF

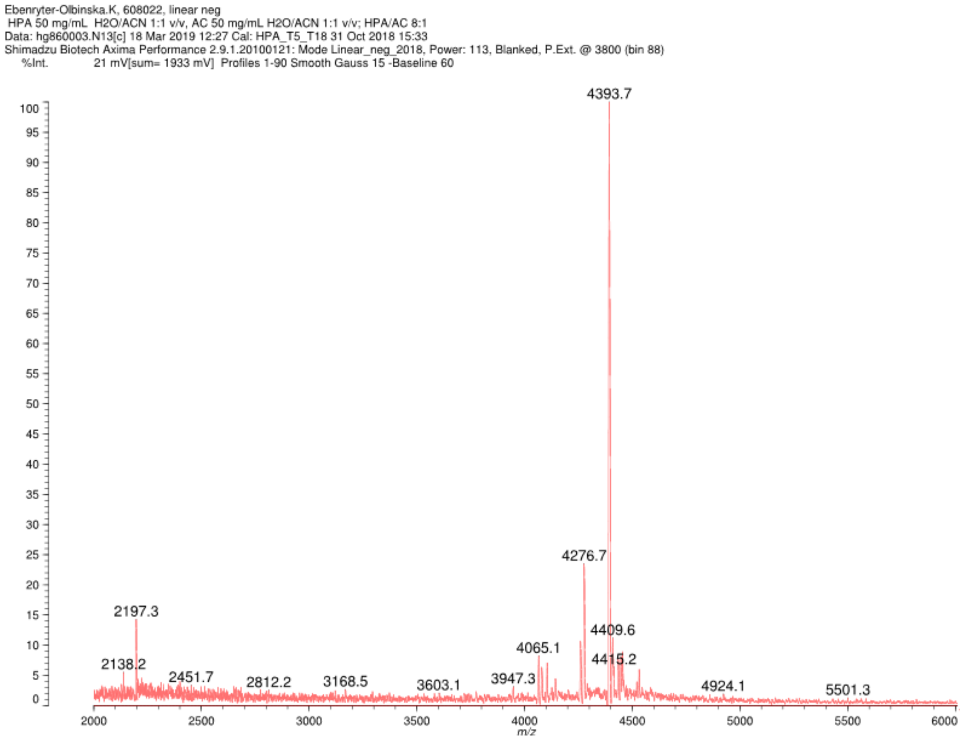

2b1: 5′-d(CTC CAG AGC CU$_{B1}$GA)-3′

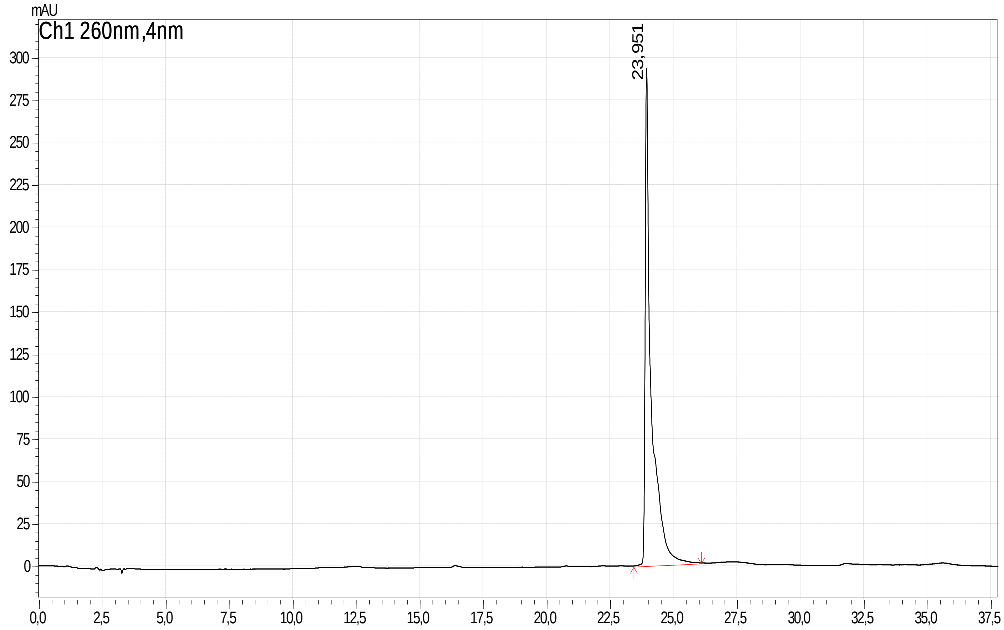

ESI-Q-TOF

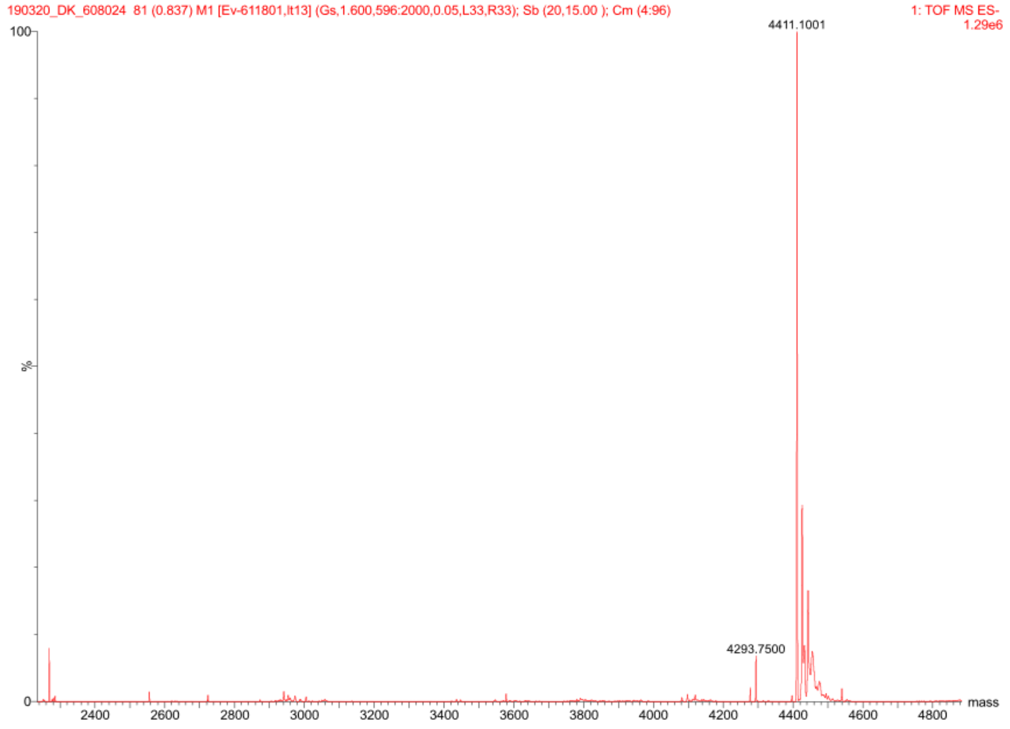

2c1: 5′-d(CUB1C CAG AGC CUB1GA)-3′

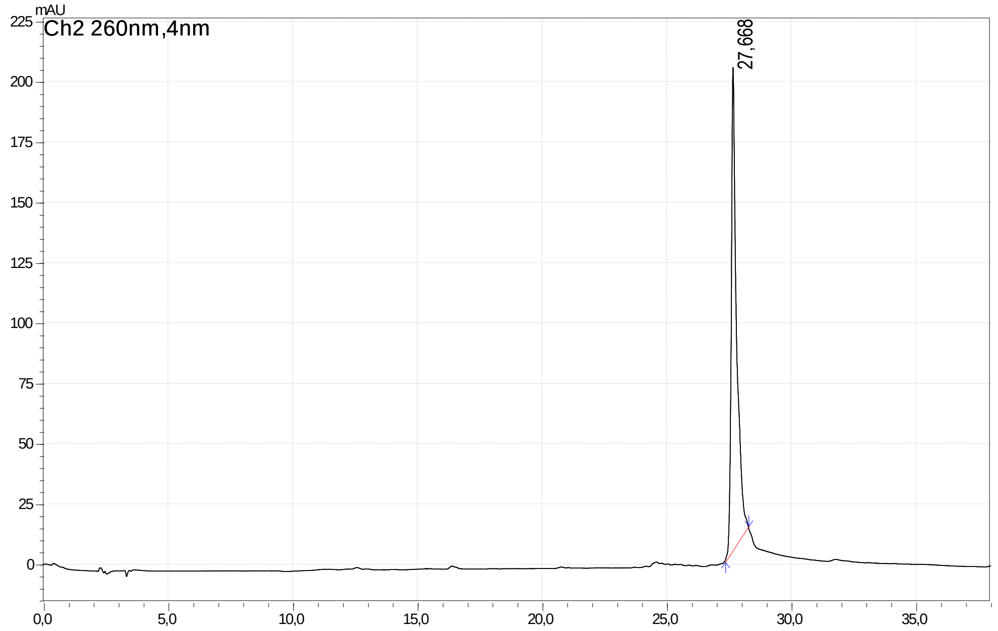

MALDI-TOF

Ebenryter-Olbinska.K, 608023, linear neg
HPA 50 mg/mL H2O/ACN 1:1 v/v, AC 50 mg/mL H2O/ACN 1:1 v/v; HPA/AC 8:1
Data: hg870003.N16[c] 18 Mar 2019 12:31 Cal: HPA_T5_T24 31 Oct 2018 15:41
Shimadzu Biotech Axima Performance 2.9.1.20100121: Mode Linear_neg_2018, Power: 116, Blanked, P.Ext. @ 3800 (bin 88)
%Int. 5.5 mV[sum= 1045 mV] Profiles 1-191 Smooth Gauss 20 -Baseline 65

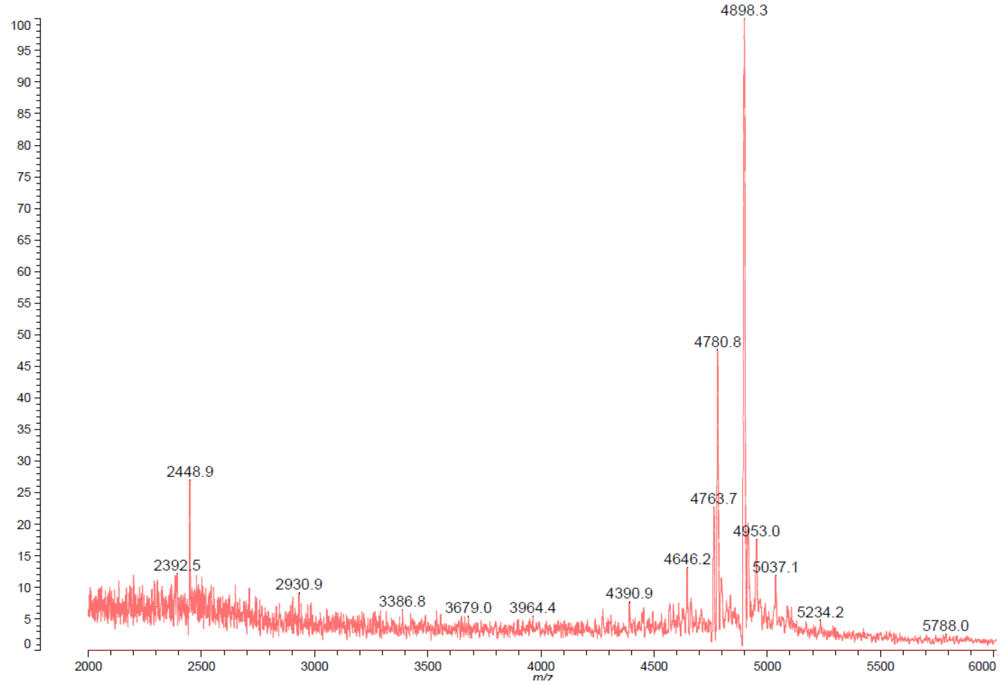

3: 5′-(GGG GGA AAA GAA A)-3′ (B3 RP-HPLC program)

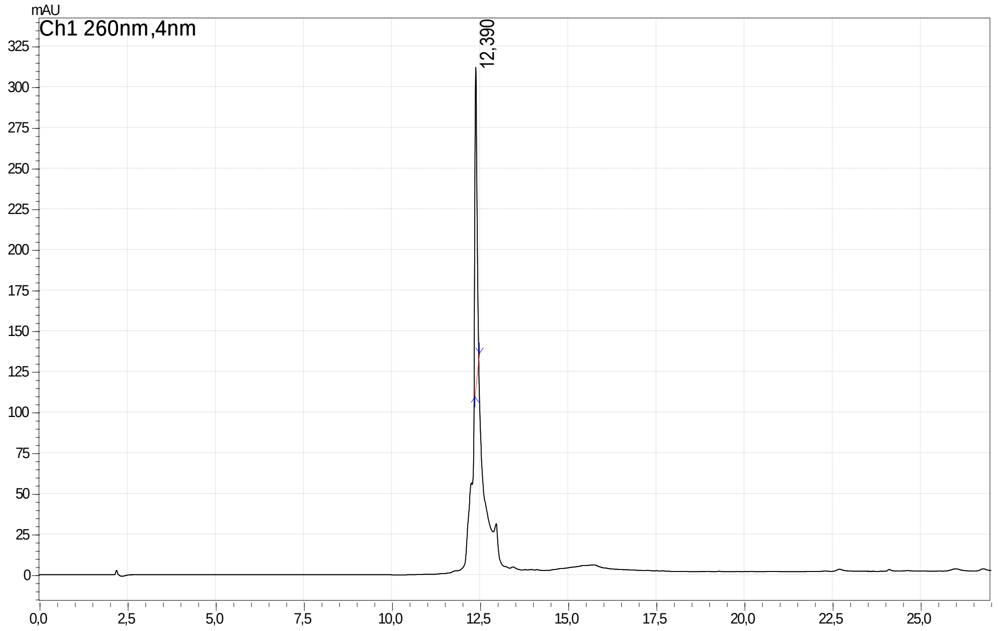

ESI-Q-TOF

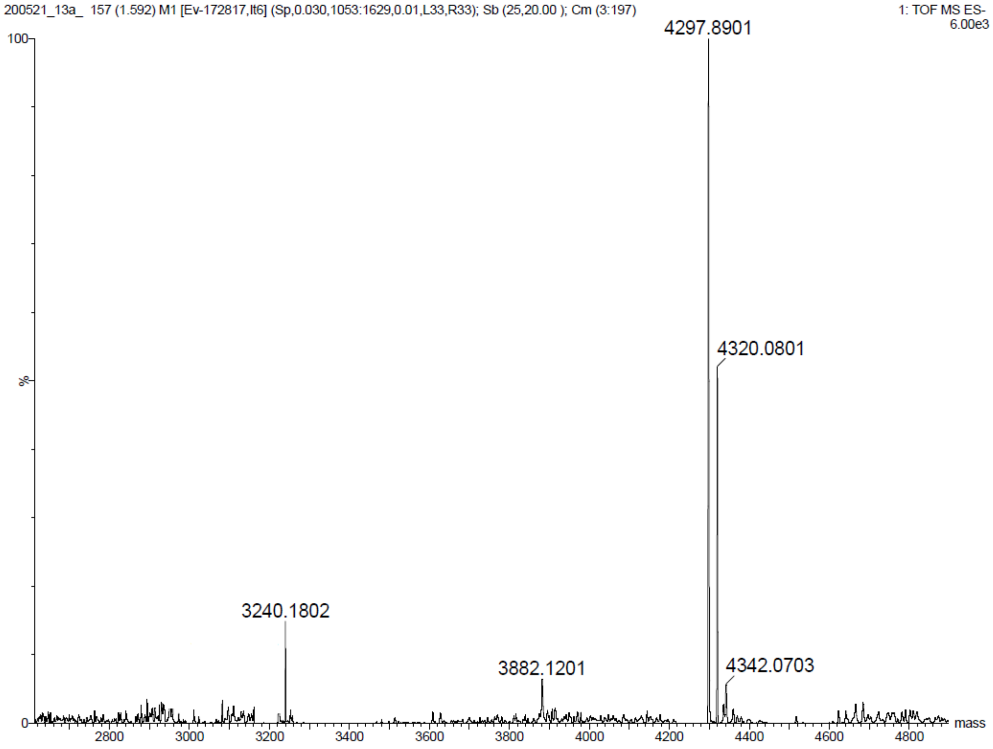

4: 5′-(UCG GGC UCU GGU G)-3′ (B3 RP-HPLC program)

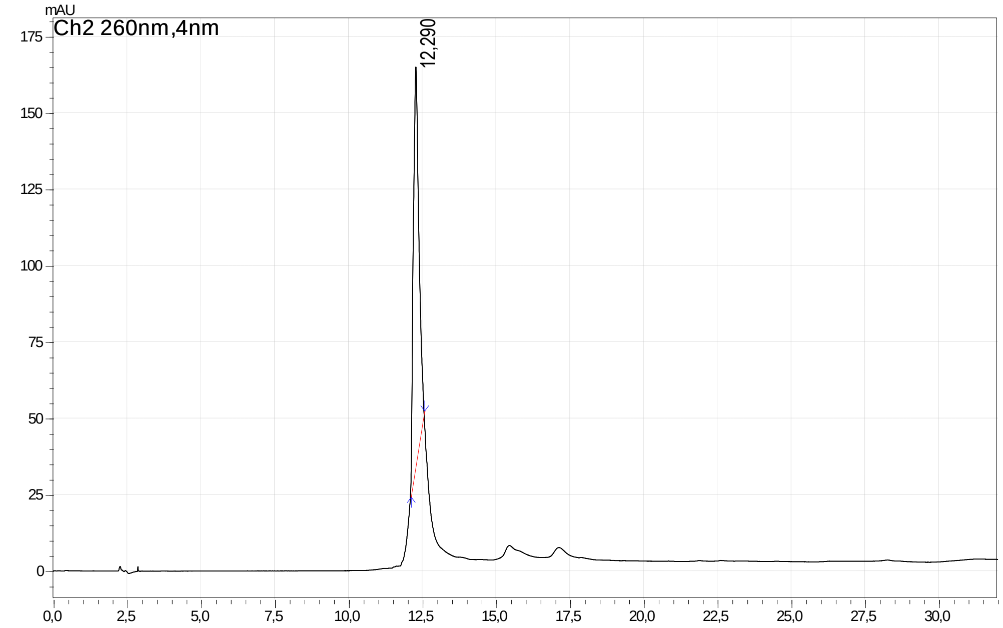

ESI-Q-TOF

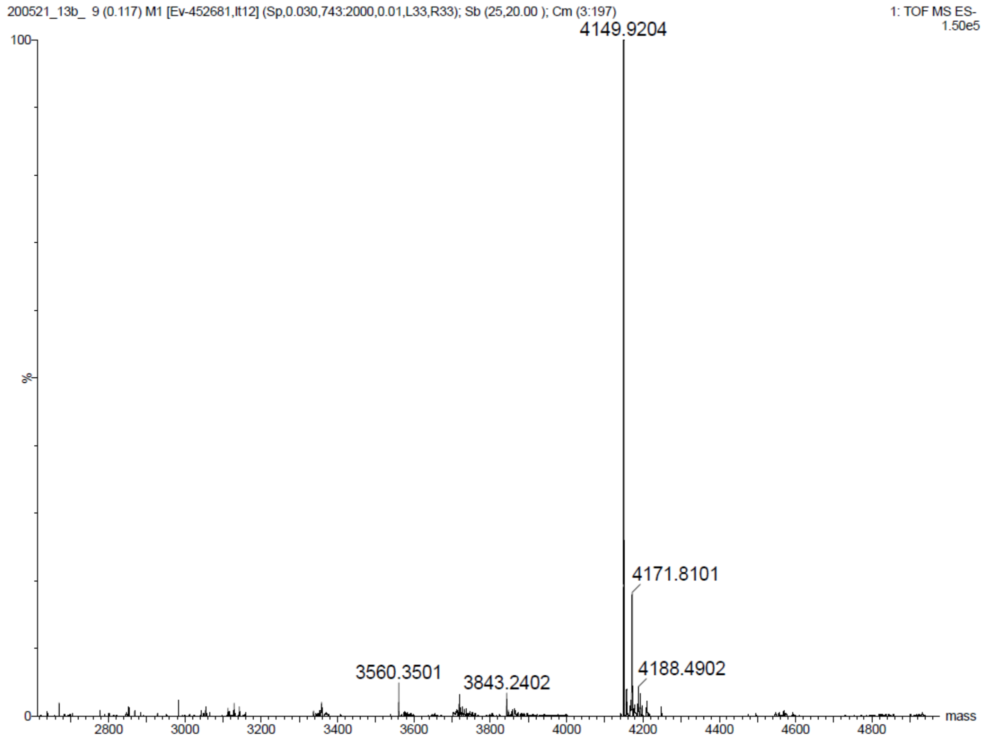

Figure S2. Spectral analysis of 8-(5-azido-3-oxa-pentoxy)-3-iron-bis(1,2-dicarbollide) $[Fe(C_2B_9H_{11})_2]^-$ (azido derivative of FESAN).

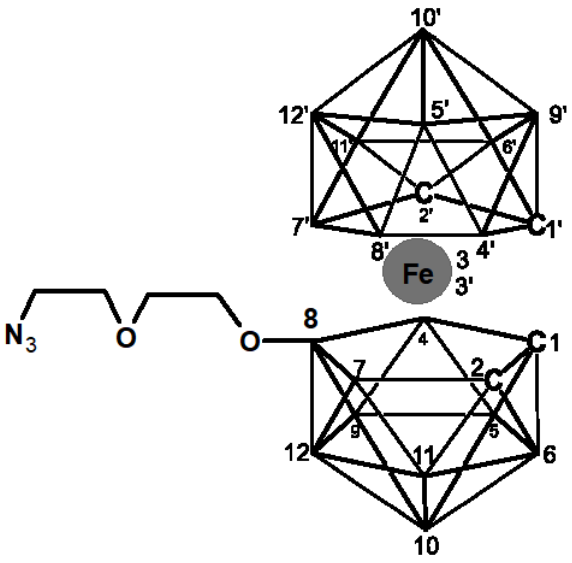

ESI-Q-TOF MS

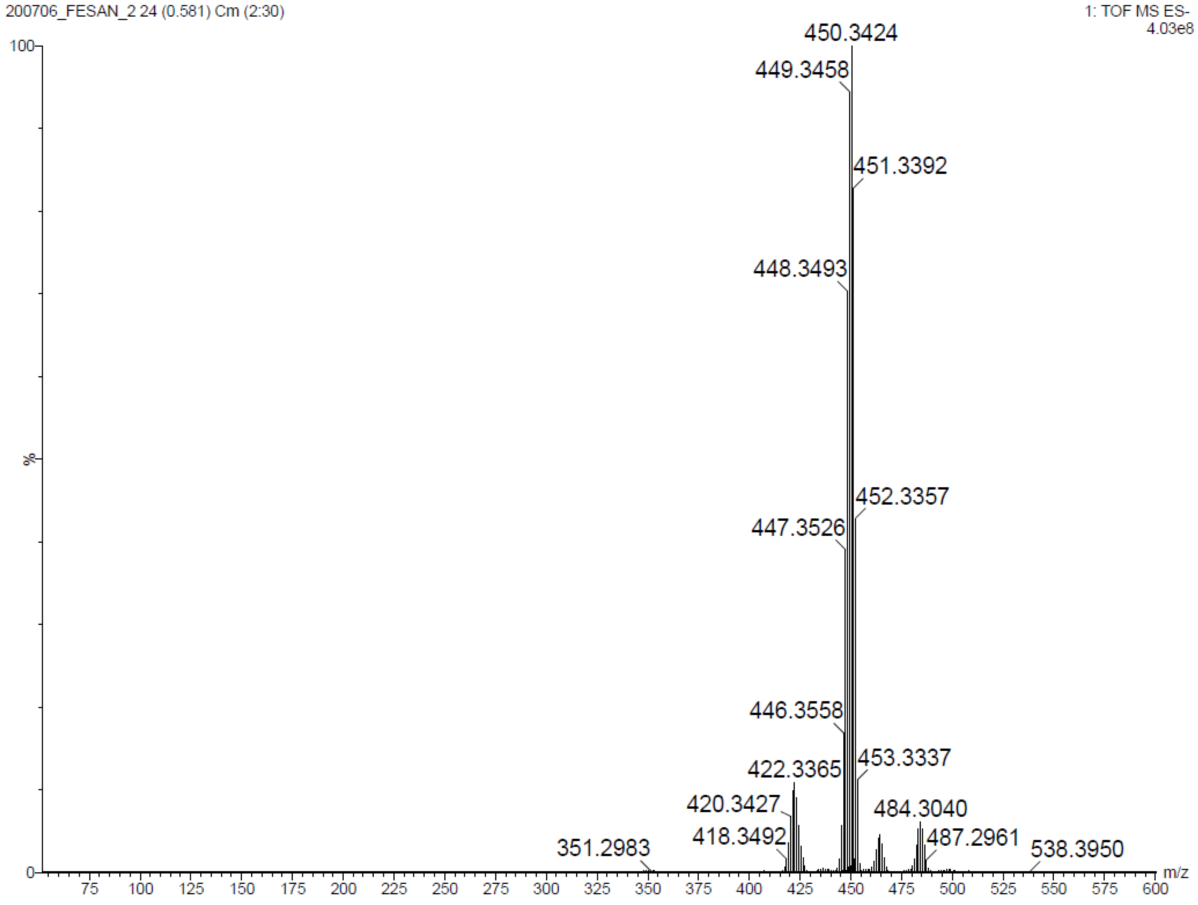

IR

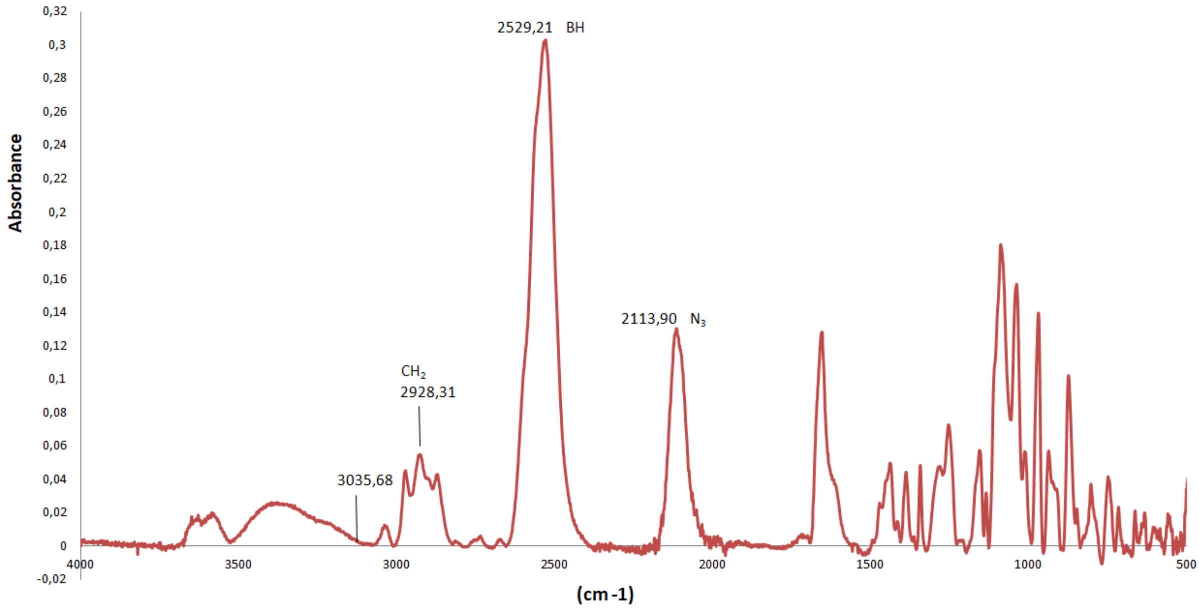

Figure S3. Spectral analysis of 1-(3-azidopropyl)-1,2-dicarba-*closo*-dodecaborane (1,2-DCDDB, [$C_2B_{10}H_{12}$]) (azido derivative of 1,2-dicarba-*closo*-dodecaborate).

^{1}H NMR

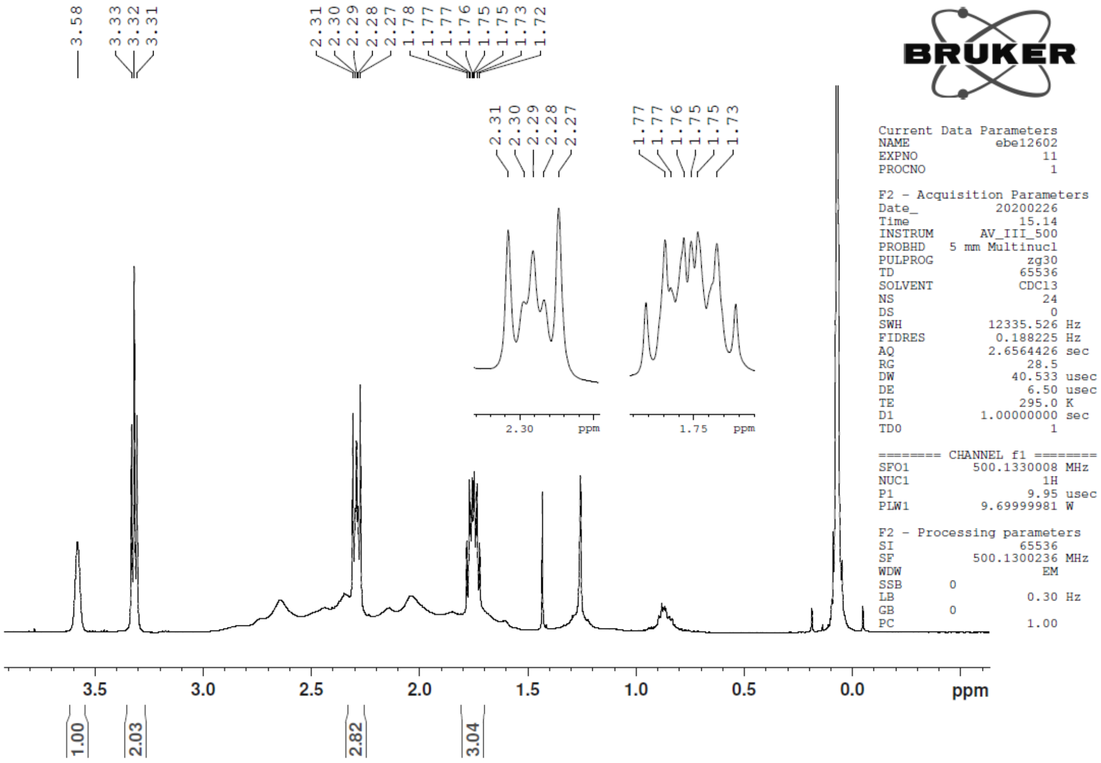

^{11}B NMR

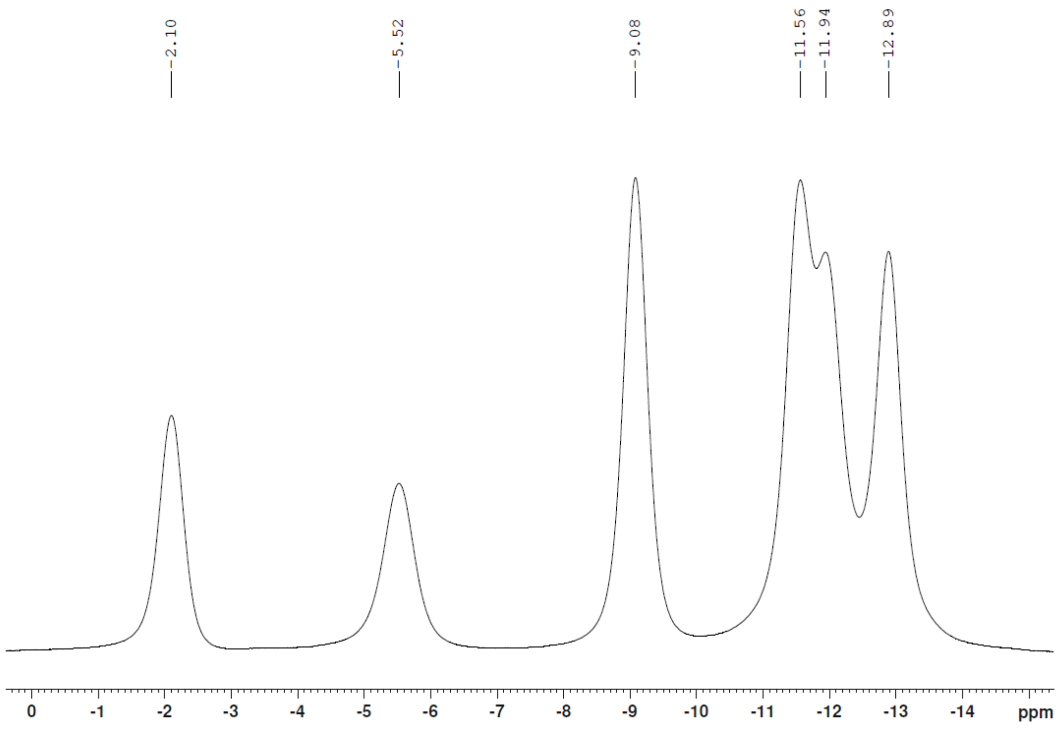

^{13}C NMR

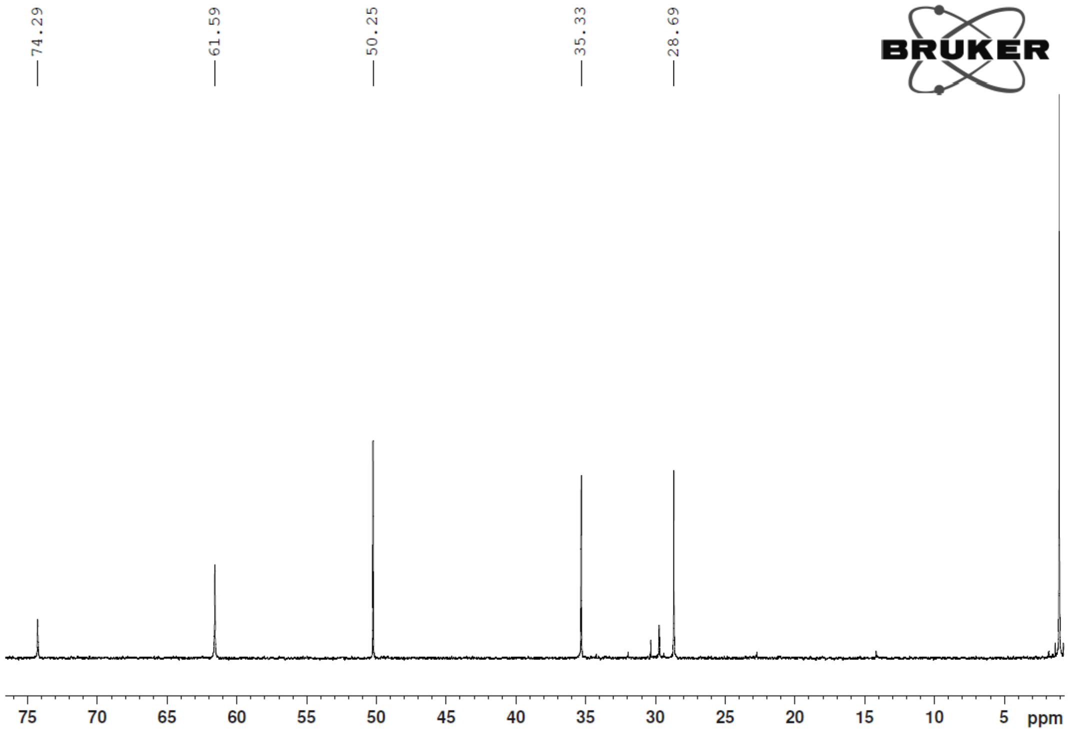

ESI-Q-TOF

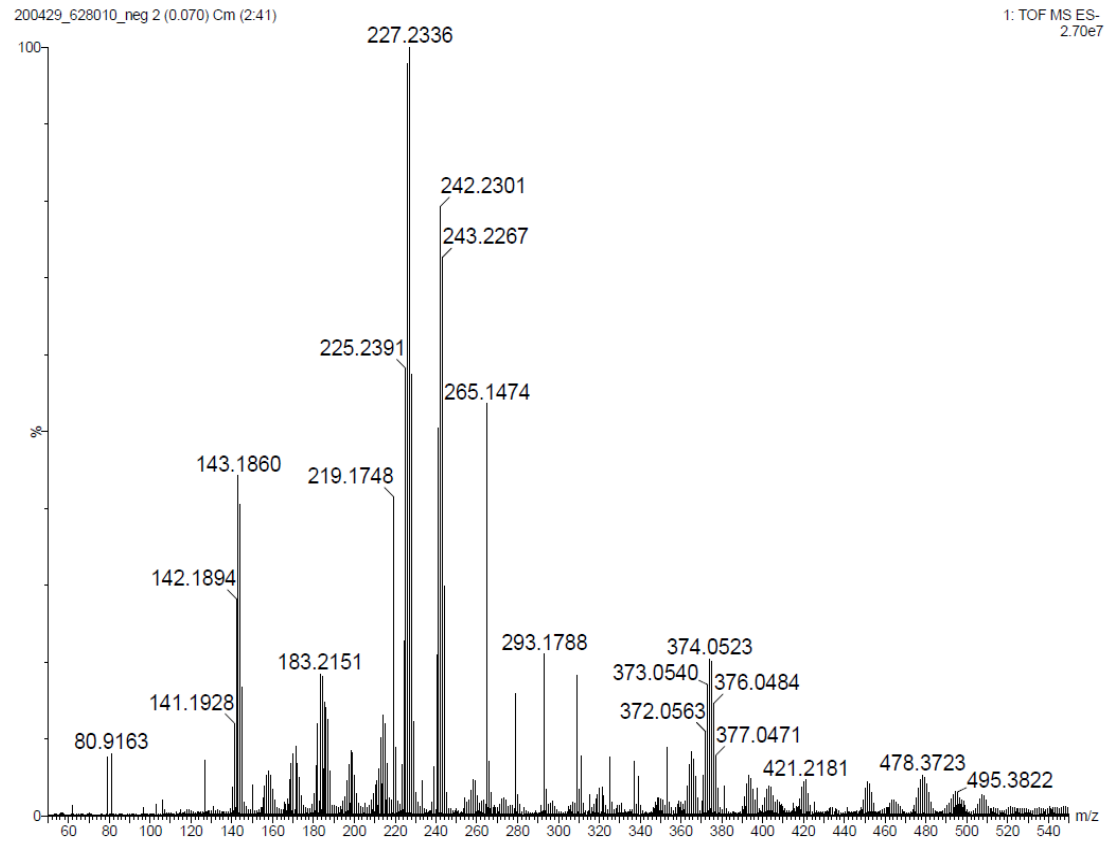
200429_628010_neg 2 (0.070) Cm (2:41)
1: TOF MS ES-
2.70e7
227.2336
242.2301
243.2267
225.2391
265.1474
143.1860
219.1748
142.1894
183.2151
293.1788
374.0523
373.0540
376.0484
141.1928
372.0563
80.9163
377.0471
421.2181
478.3723
495.3822
100
%
0
m/z

IR

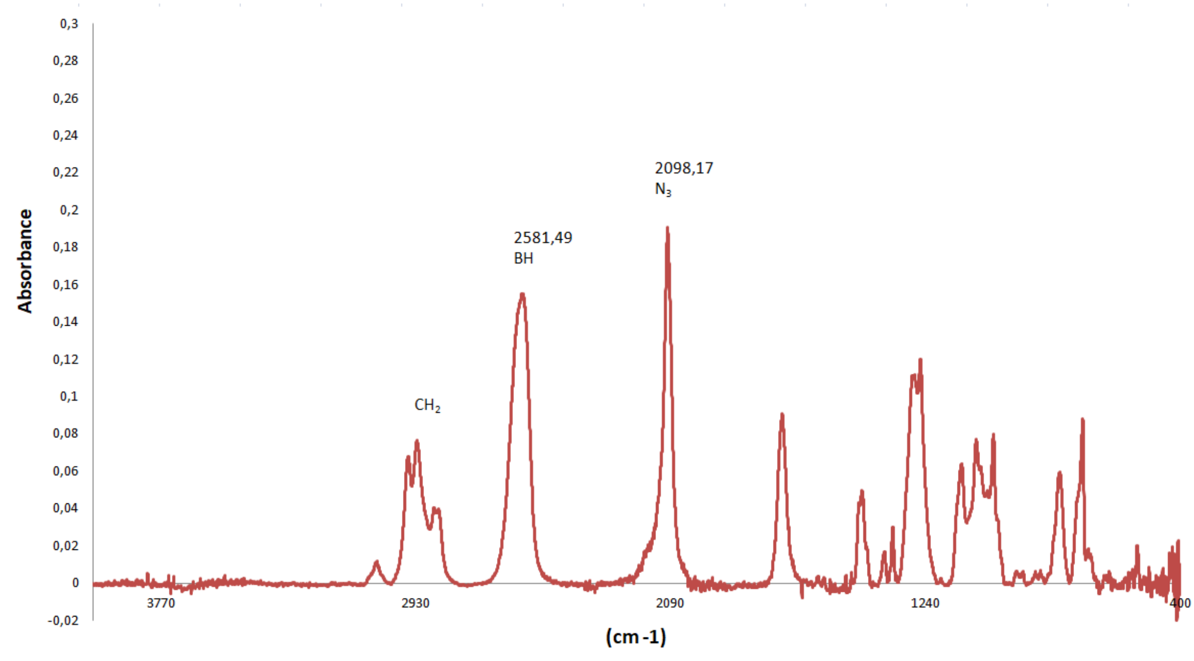
Absorbance
2098,17
N3
2581,49
BH
CH2
3770
2930
2090
1240
400
(cm -1)

Figure S4. Spectral analysis of the bis-tetrabutylammonium-(4-azidobuthoxy)-undecahydro-*closo*-dodecaborate (DDB, $[B_{12}H_{12}]^{2-}$) (azido derivative of *closo*-dodecaborate).

^{1}H NMR

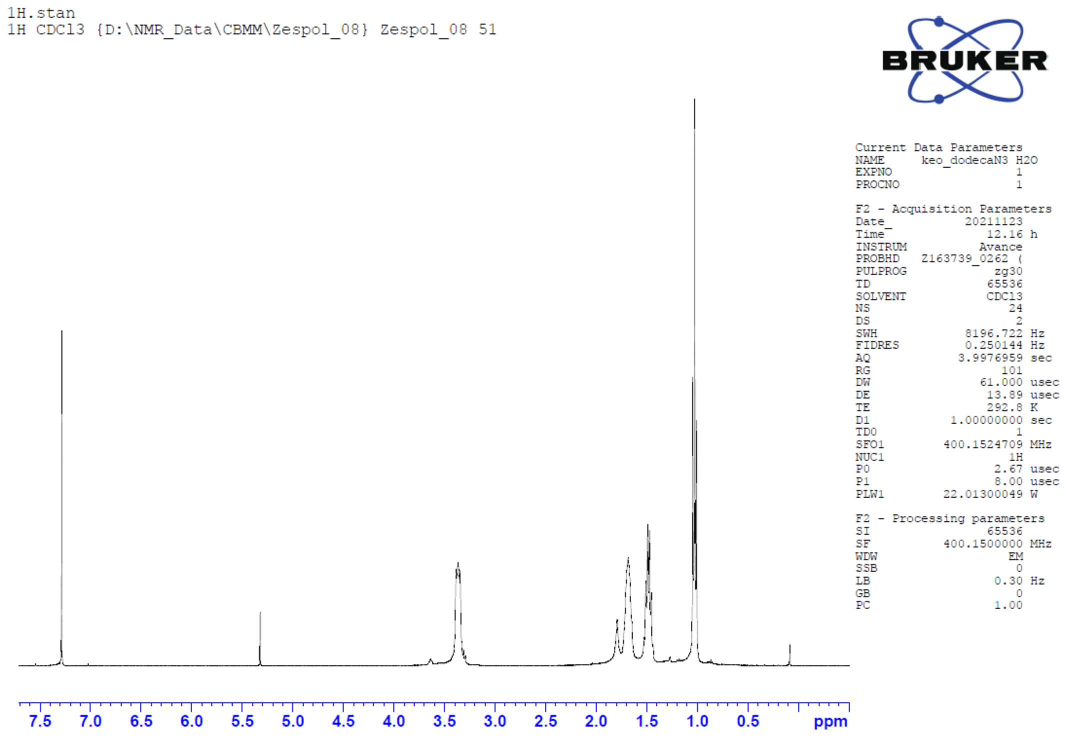

^{13}C NMR

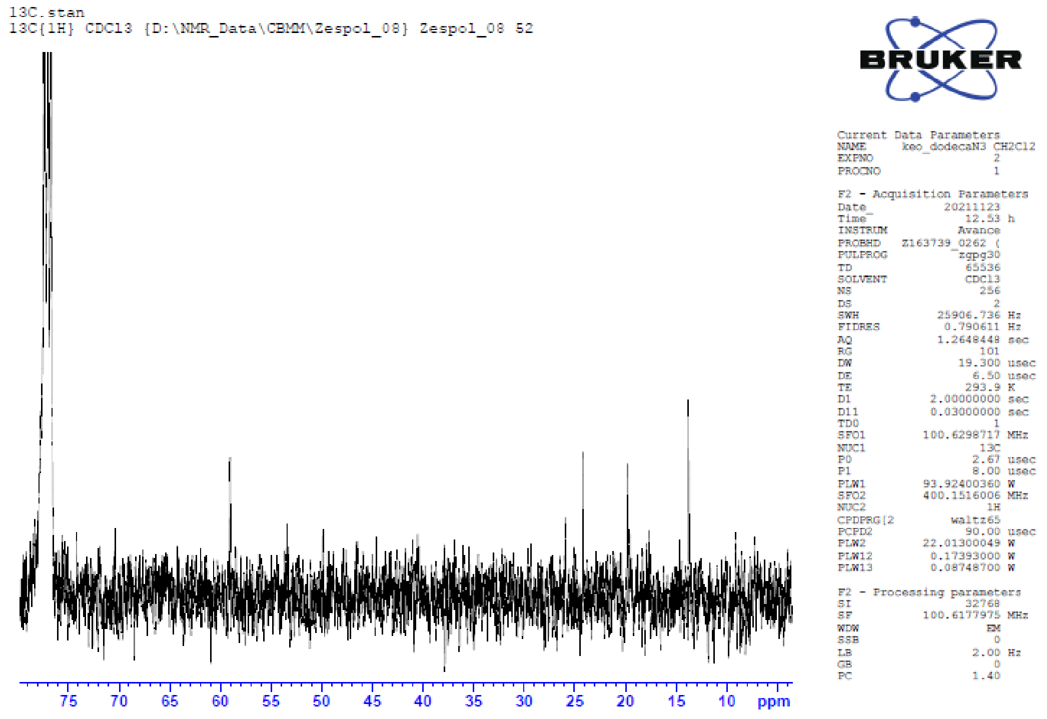

^{11}B NMR

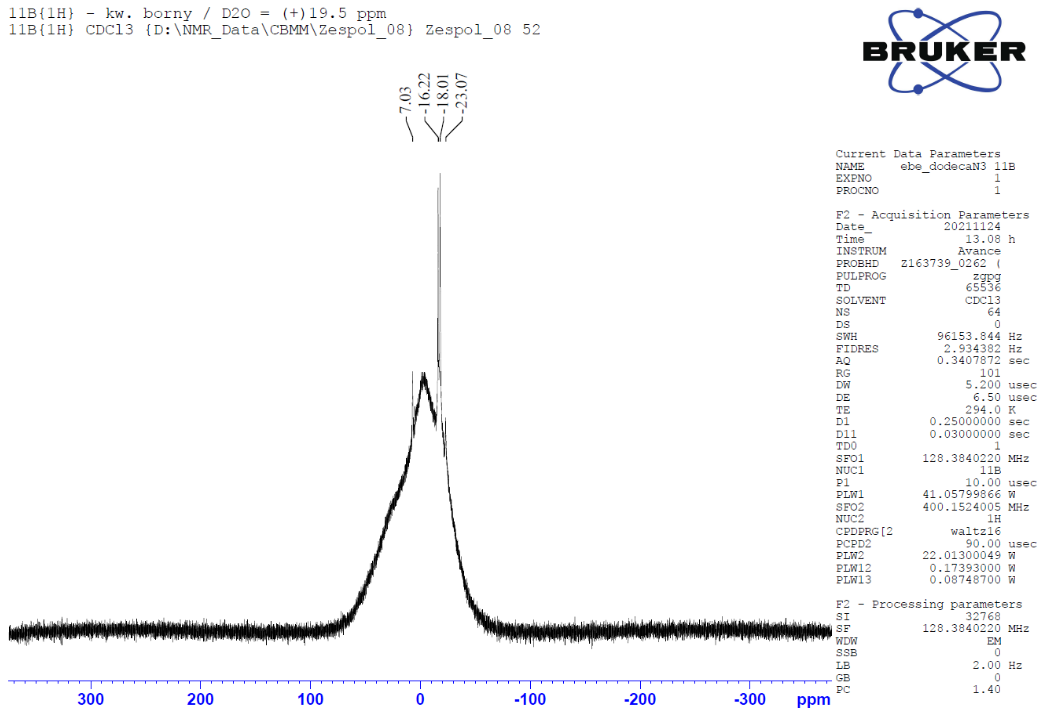

11B{1H} - kw. borny / D2O = (+)19.5 ppm
11B{1H} CDCl3 {D:\NMR_Data\CBMM\Zespol_08} Zespol_08 52
7.03
-16.22
-18.01
-23.07
Current Data Parameters
NAME ebe_dodecaN3 11B
EXPNO 1
PROCNO 1
F2 - Acquisition Parameters
Date_ 20211124
Time 13.08 h
INSTRUM Avance
PROBHD Z163739_0262 (
PULPROG zgpg
TD 65536
SOLVENT CDCl3
NS 64
DS 0
SWH 96153.844 Hz
FIDRES 2.934382 Hz
AQ 0.3407872 sec
RG 101
DW 5.200 usec
DE 6.50 usec
TE 294.0 K
D1 0.25000000 sec
D11 0.03000000 sec
TD0 1
SFO1 128.3840220 MHz
NUC1 11B
P1 10.00 usec
PLW1 41.05799866 W
SFO2 400.1524005 MHz
NUC2 1H
CPDPRG[2 waltz16
PCPD2 90.00 usec
PLW2 22.01300049 W
PLW12 0.17393000 W
PLW13 0.08748700 W
F2 - Processing parameters
SI 32768
SF 128.3840220 MHz
WDW EM
SSB 0
LB 2.00 Hz
GB 0
PC 1.40
300
200
100
0
-100
-200
-300
ppm

IR

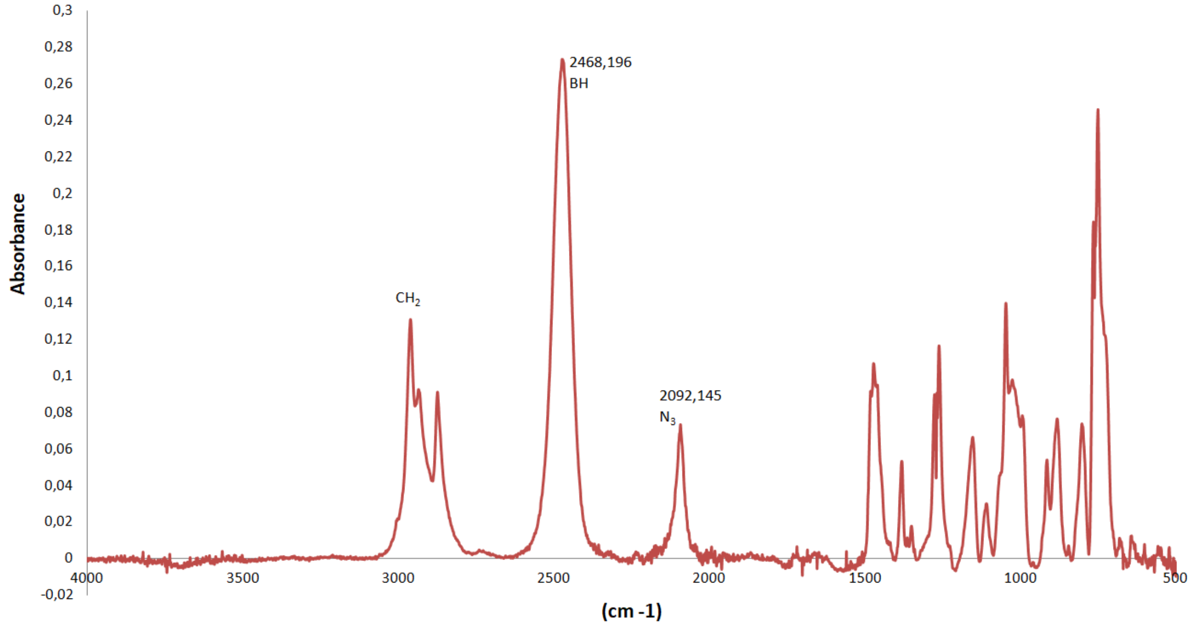

Absorbance
0,3
0,28
0,26
0,24
0,22
0,2
0,18
0,16
0,14
0,12
0,1
0,08
0,06
0,04
0,02
0
-0,02
4000
3500
3000
2500
2000
1500
1000
500
(cm -1)
CH2
2468,196
BH
2092,145
N3
